# Hepatocellular carcinoma (HCC) tumor microenvironment is more suppressive than colorectal cancer liver metastasis (CRLM) tumor microenvironment

**DOI:** 10.1007/s12072-023-10537-6

**Published:** 2023-05-04

**Authors:** Sara Santagata, Giuseppina Rea, Daniela Castaldo, Maria Napolitano, Anna Capiluongo, Crescenzo D’Alterio, Anna Maria Trotta, Caterina Ieranò, Luigi Portella, Salvatore Di Maro, Fabiana Tatangelo, Vittorio Albino, Rita Guarino, Carmen Cutolo, Francesco Izzo, Stefania Scala

**Affiliations:** 1 Microenvironment Molecular Targets, Istituto Nazionale Tumori-IRCCS-Fondazione “G. Pascale”, Via Semmola, 80131 Naples, Italy; 2 Department of Environmental, Biological and Pharmaceutical Sciences and Technologies, University of Campania “Luigi Vanvitelli”, Via Vivaldi 43, 81100 Caserta, Italy; 3 Pathology, Istituto Nazionale Tumori-IRCCS-Fondazione “G. Pascale”, Via Semmola, 80131 Naples, Italy; 4 Divisions of Hepatobiliary Surgery, Istituto Nazionale Tumori-IRCCS-Fondazione “G. Pascale”, Via Semmola, 80131 Naples, Italy

**Keywords:** Liver cancer, Liver microenvironment, Inflammation-associated cancer, Liver metastases, Immune cells and the microenvironment, Regulatory T cells, Myeloid-derived suppressor cells, ENTPD1, CXCR4, CXCR4 inhibitors

## Abstract

**Background and purpose:**

While HCC is an inflammation-associated cancer, CRLM develops on permissive healthy liver microenvironment. To evaluate the immune aspects of these two different environments, peripheral blood-(PB), peritumoral-(PT) and tumoral tissues-(TT) from HCC and CRLM patients were evaluated.

**Methods:**

40 HCC and 34 CRLM were enrolled and freshly TT, PT and PB were collected at the surgery. PB-, PT- and TT-derived CD4^+^CD25^+^ Tregs, M/PMN-MDSC and PB-derived CD4^+^CD25^−^ T-effector cells (Teffs) were isolated and characterized. Tregs’ function was also evaluated in the presence of the CXCR4 inhibitor, peptide-R29, AMD3100 or anti-PD1. RNA was extracted from PB/PT/TT tissues and tested for FOXP3, CXCL12, CXCR4, CCL5, IL-15, CXCL5, Arg-1, N-cad, Vim, CXCL8, TGFβ and VEGF-A expression.

**Results:**

In HCC/CRLM-PB, higher number of functional Tregs, CD4^+^CD25^hi^FOXP3^+^ was detected, although PB-HCC Tregs exert a more suppressive function as compared to CRLM Tregs. In HCC/CRLM-TT, Tregs were highly represented with activated/ENTPD-1^+^Tregs prevalent in HCC. As compared to CRLM, HCC overexpressed CXCR4 and N-cadherin/vimentin in a contest rich in arginase and CCL5. Monocytic MDSCs were highly represented in HCC/CRLM, while high polymorphonuclear MDSCs were detected only in HCC. Interestingly, the function of CXCR4-PB-Tregs was impaired in HCC/CRLM by the CXCR4 inhibitor R29.

**Conclusion:**

In HCC and CRLM, peripheral blood, peritumoral and tumoral tissues Tregs are highly represented and functional. Nevertheless, HCC displays a more immunosuppressive TME due to Tregs, MDSCs, intrinsic tumor features (CXCR4, CCL5, arginase) and the contest in which it develops. As CXCR4 is overexpressed in HCC/CRLM tumor/TME cells, CXCR4 inhibitors may be considered for double hit therapy in liver cancer patients.

**Supplementary Information:**

The online version contains supplementary material available at 10.1007/s12072-023-10537-6.

## Introduction

Liver cancer accounts for 8.3% of global cancer deaths with 905,677 new cases worldwide [1]. The two most common liver-established tumors are hepatocellular carcinoma (HCC) and colorectal cancer liver metastasis (CRLM). HCC accounts for 75–85% of primary liver cancers and is mainly HBV/HCV related. Metabolic or inherited disorders causing chronic inflammation, fibrosis and/or dysregulated hepatic regeneration are responsible for tumor promotion [2]. The liver harbors the largest number of immune cells in the body and maintains a unique immune state, more tolerant than other organs due to the constant flow of inflammatory signals from the gut. HCC is a typical inflammation-dependent cancer where immune infiltrates are associated with better prognosis [3]. As IL-6, lymphotoxin-α and TNF can accelerate hepatocarcinogenesis and affect tumor proliferation and invasion [4], immune responses limit liver cancer progression [3]. Inflammation triggers the de-differentiation of liver cells into tumorigenic stem-like cells. These phenomena explain the intra- and intertumor heterogeneity existing among different types of HCC and are associated with tumor microenvironment (TME) evolution toward increasing immunosuppressive phenotype [5]. Lesions in the same tumor may have distinctive genomic alterations, biological behaviors and local microenvironments and can respond differently to therapies, but the immune status of the HCC microenvironment was relatively less heterogenous recognizing three distinctive HCC-TME subtypes (immunocompetent, immunodeficient and immunosuppressive) strongly dependent on T regulatory cells (Tregs) infiltration [6].

Liver TME is composed of Tregs, natural killer cells (NKs), tumor-associated macrophages (TAMs), myeloid-derived suppressor cells (MDSCs), non-immune cells such as hepatic stellate cells (HSCs) and liver sinusoidal endothelial cells (LSECs) [7–9]. Among the major protumorigenic mechanisms, the secretion of cytokines and growth factors favor proliferation or counteract apoptosis of tumor cells as well as suppressing the antitumor function of neighboring lymphocytes [10]. Tregs are considered a major culprit in mediating T cell dysfunction in HCC through the secretion of IL-10 and TGFß [11, 12]. Overall, the immune microenvironment in the liver is dominated by immunosuppressive cells (Kupffer cells monocyte-derived macrophages and MDSCs) and signals (VEGF, TGFβ and arginase), which suppress T cell activation [3, 13]. The tumor-derived cytokines CXCL5 and CCL15 recruit immunosuppressive neutrophils and monocytes [14, 15]. Conversely, proinflammatory cytokines, such as IL-2, IFNγ, CXCL10 and CXCL9, attract cells to mount antitumor immune response [3, 16] and the balance of these stimuli defines the quality of the immune composition and response. While HCC develops on TME of liver chronic inflammation, [2] CRLM surrounding tissue is healthy liver [17] colonized by pre-metastatic and posttumor invasion niche [18]. Colorectal cancer (CRC) is the fourth cause of cancer deaths worldwide [1], with metastasis the critical cause of CRC-related death [13]. CRLM represents the distant metastatic disease for 50% of CRC patients [19] occurring as synchronous or after primary tumor removal. A small subset of CRC cells evade from the primary CRC undergoing morphological changes such as epithelial-to-mesenchymal transition (EMT), migration through the extracellular matrix (ECM) and invasion into the neighboring tissues [13]. In addition, hepatic TME, with macrophages, T cells, B cells, cytokines, chemokines and exosomes plays a crucial role. A suppressive immunologic microenvironment, characterized by TAMs and Tregs, plays a tumor-promoting role for CRLM. TAMs maintain the immunosuppressive environment through PDL1, PDL2 and other inhibitory receptors, while activating Treg cells by secreting IL-10 and TGFβ [20], but also releasing a plethora of ECM remodeling factors and matrix metalloproteases (MMPs) [18, 21]. Conversely, protective Tregs were also described in CRC with reduced MMPs in patients with higher ratio of intratumoral Tregs/IL-17 producing T cells [21, 22]. The chemokine receptor CXCR4 is overexpressed in HCC [23] in primary and metastatic colorectal cancer [24, 25]. CRC-CXCR4 promotes CXCL12/SDF-1 secretion by HSC that stimulates CRC-TGFβ secretion, promoting liver metastasis [26]. In HCC patients, high CXCR4 levels correlated with an unfavorable prognosis for overall survival [23]. High CXCR4 and Mut-KRAS identify the worst prognostic group within a homogeneous cohort of neoadjuvant-treated, metastatic CRC patients with resectable liver metastases [27]. Highly selective, small molecule CXCR4 antagonist was reported to suppress tumor growth, prevent distant metastasis and tumor-associated macrophage infiltration in HCC [28]. Targeting CXCR4 also impairs the recruitment of Tregs in murine and human cancers [29–31]. With the intent to characterize the role of TME on primary and secondary HCC lesions, Tregs were evaluated in 40 HCC and 34 CRLM and compared to Tregs in peripheral blood and peritumoral tissues. Moreover, the possible therapeutic CXCR4 inhibition was evaluated ex vivo on patients’ Tregs.

## Materials and methods

### Cell culture

Huh7 (human hepatoma cell line) were kindly provided by Dr. Steven A Curley (Surgical Oncology, CHRISTUS Mother Frances Hospital, Tyler, Texas, USA) and cultured in Iscove’s modified Dulbecco’s medium (IMDM) supplemented with 2-mM L-glutamine, 100 U/ml penicillin, 100 μg streptomycin, and 10% fetal bovine serum. HT-29 (human colon cancer cell lines) were cultured in RPMI-1640 Medium supplemented with 2-mM L-glutamine, 100 U/ml penicillin, 100 μg streptomycin, and 10% fetal bovine serum (GE Healthcare Life Sciences, HyClone Laboratories). Huh7 is a permanent cell line established from male hepatoma tissue, surgically removed from a 57-year-old Japanese male in 1982 [32]. Short tandem repeat (STR) profile of 16 loci indicates the absence of Y chromosome. Chromosome number varies (55–63) with 60 chromosomes in less than 1/3 of the population, indicating heterogeneous cell populations. Abnormalities are detected in all chromosomes except for chromosome 21. M-FISH revealed 32 common abnormalities consisting of trisomy 20, loss of Y, 6 partial intra-chromosomal gains or losses, and 24 inter-chromosomal rearrangements [33]. Newly identified variants in Huh7 cell line (SNV 4,094; insertion 911; deletion 1,898) were reported. To establish efficient deletions characteristic of Huh7 cells, PCR primer sets were designed using the dataset of homozygous deletions identified in whole-genome sequencing [34]. Moreover, Huh7 cell line displayed TP53 and PREX2 missense mutation; TERT promoter mutation; FGF19 and CCND1 focal amplification; KMT2D truncation mutation [35].

### Patients and specimens

74 patients, HCC (*n* = 40) and CRLM (*n* = 34), eligible for surgical resection were enrolled at the Hepatobiliary Surgical Oncology Division of Istituto Nazionale per lo Studio e la Cura dei Tumori, Fondazione “G. Pascale” in Naples. In the majority of HCC patients, a viral etiology was found [32/40 (80%): 29/32 HCV; 2/32 HBV and 1/32 both HBV/HCV positive], in five (12%) patients a non-viral etiology, and for three (8%) patients the information was missing. 31/40 (78%) patients exhibited hepatic cirrhosis and 36/40 (90%) chronic hepatitis; 33/40 (82%) exhibited a moderately differentiated tumor grade (G2), 4/40 (10%) a poorly differentiated tumor grade (G3) and for 3/40 (8%) info on grading was missing. Out of CRLM patients, 13/34 (38%) presented single or 20/34 (59%) multiple nodules, while for 1/34 (3%) information was missing. For the majority of patients, 26/34 (76%) showed a moderately differentiated tumor grade (G2), 1/34 (3%) a poorly differentiated tumor grade (G3), and for 7/34 (21%) information on grading was missing. HCC and CRLM patient’s features are shown in Table 1 and Supplementary Tables 1, 2. 8 mL of heparinized peripheral blood (PB) was collected before surgery. Heparinized blood was collected from 30 healthy donors (HD). In addition, freshly isolated HCC/CRLM tumor tissue (TT) and the corresponding normal-appearing tissue, called peritumoral tissue (PT), sample specimens were collected at the time of surgery. 3 cm was used as the minimal distance between the TT and PT samples. Peripheral blood mononuclear cells (PBMCs) were obtained from patients and HD through Ficoll–Hypaque density gradient centrifugation (GE Healthcare Bioscience). TT and PT samples derived from patients were minced into small pieces and digested with 1 mg/mL of collagenase (Sigma-Aldrich) for 30 min at 37 °C, transferred to a cell strainer (70 µm Nylon) (BD Biosciences), and gently separated through a syringe plug to isolate tissue-infiltrating lymphocytes. Tregs from PB, PT, and TT were immediately used for the experiments.

**Table 1:** Patients characteristics

	HCC (*n* = 40)	CRLM (*n* = 34)
	*N* (%)	*N* (%)
Age (median years)		
< 66	12 (30)	25 (73)
≥ 66	28 (70)	9 (27)
Gender		
Male	28 (70)	20 (59)
Female	12 (30)	14 (41)
Number of nodules		
1	30 (75)	13 (38)
2	9 (22)	6 (17)
3	–	7 (21)
> 3	–	7 (21)
Unknown	1 (3)	1 (3)
Size of nodules (cm)		
< 3	11 (28)	17 (50)
≥ 3	27 (67)	17 (50)
Unknown	2 (5)	–
Etiology		
HCV	29 (72)	–
HBV	2 (5)	–
Both HCV and HBV	1 (3)	–
Non-viral	5 (12)	–
Unknown	3 (8)	–
Chronic hepatitis		
Yes	36 (90)	–
No	1 (3)	–
Unknown	3 (7)	–
Hepatic cirrhosis		
Yes	31 (78)	–
No	6 (15)	–
Unknown	3 (7)	–
Child–Pugh		
A	23 (57)	–
B	5 (13)	–
Unknown	12 (30)	–
Grading		
G2	33 (82)	26 (76)
G3	4 (10)	1 (3)
Unknown	3 (8)	7 (21)
CEA level (ng/mL)		
< 47	–	23 (67)
≥ 47	–	4 (12)
Unknown	–	7 (21)
Primary T category		
T1–T2	–	7 (21)
T3–T4	–	21 (62)
Unknown	–	6 (17)
Primary N category		
N0	–	8 (23)
N1–N2	–	19 (56)
Unknown	–	7 (21)

### Flow cytometry

Flow cytometry was performed on venous PB samples collected in heparin-coated vacutainer tubes, and on PT and TT cell suspension using a FACS Aria III flow cytometer, daily settled with Calibrite (Fitc, Pe, PerCP and APC) and Compbeads (Pe-Cy7 and APC-Cy7; Becton Dickinson, San Jose, CA, USA) beads. Fluorochrome-labeled monoclonal antibodies (BD Bioscience) were used: Fitc-anti-FOXP3 (clone 259D/C7), Pe-Cy7-anti-CD25 (clone 2A3), APC-Cy7-anti-CD4 (clone RPA-T4), APC-anti-CD45RA (clone HI100), Pe-anti-CD152 (CTLA-4) (clone BNI3), PercP-anti-CD184 (CXCR4) (clone 12G5), APC-anti-CD279 (PD-1) (clone MIH4), Pe-anti-CD278 (ICOS) (clone DX29) and APC-anti-CD39 (ENTPD1) (clone TU66) for identification and characterization of PB-, PT- and TT-derived Tregs; Fitc-anti-lineage 1 (NCAM 16.2; MφP9; 3G8; SJ25C1; L27; SK7), Pe-anti-CD11b (clone ICRF44), PercP-anti-CD33 (clone WM-53), Pe-Cy7 anti-HLA-DR (clone G46-6), APC-anti-CD15 (clone HIM1), and APC-Cy7 anti-CD14 (clone MφP9) for MDSCs subsets identification; Pe-anti-CD8 (clone SK1), APC-anti-CD3 (clone UCHT1), APC-anti-CD45RA (clone HI100) and FITC-anti-CD62L (clone SK11) for identification of effector CD8^+^T cells. FOXP3 and CTLA-4 protein detection was performed using a fixation and permeabilization commercially available kit (Transcription Factor Buffer Set, BD Pharmingen) according to the manufacturer’s instructions. A minimum of 100.000 events for each sample was collected and data analyzed using FacsDiva software 8.01 (BD Bioscience).

### Ki67 detection

7.5 × 10^4^ Huh7 or HT-29 cells were seeded in 1 mL of complete growth medium in 24-well and cultured for 24 h until they had fully adhered. PBMCs from HCC or CRLM patients or HD were stimulated 16 h with IL-2 (50 ng/mL) (Miltenyi Biotec) in complete medium. Subsequently, PBMCs (7.5 × 10^5^ cells/well) were added to tumor cells in a final volume of 2 mL (10:1 ratio) and incubated at 37 °C and 5% CO2 for 48 h. After 48 h, PBMCs were eliminated and adherent Huh7 or HT-29 cells were detached. Cell viability was analyzed by 7-AAD staining (Miltenyi Biotec). After fixation and permeabilization with commercially available kit (Transcription Factor Buffer Set, BD Pharmingen), tumor cells were stained with PE-anti-Ki67 antibody (BD Bioscience, San Jose, CA, USA). The samples were analyzed by flow cytometry.

### Purification of T cell subsets

PB-, PT- and TT-derived CD4^+^CD25^+^ Tregs and PB-derived CD4^+^CD25^−^ T-effector cells (Teffs) were isolated using the Dynabeads Regulatory CD4^+^CD25^+^ T cell kit (> 95% purity tested by flow cytometry). Briefly, CD4^+^ cells were isolated by negative selection, then a depletion beads solution was added to remove the non-CD4^+^ cells. CD25 beads were added to CD4^+^ T cells to capture the CD4^+^CD25^+^ Tregs and the remaining fraction corresponded to CD4^+^CD25^−^ Teff cells. All purification steps were performed according to the manufacturer’s instructions (Invitrogen by Life Technologies).

### Suppression assay of Tregs

Carboxyfluorescein diacetate succinimidyl ester (CFSE)-labeled autologous PB-derived Teffs (CellTrace CFSE Cell Proliferation Kit, Molecular Probes, by Life Technologies) were cultured with PB-derived Tregs at different ratios (1:0, 1:1, 1:0.5, 1:0.25, 1:0.125, 1:0.062, 1:0.031, 1:0.015 and 1:0.007) and with TT- or PT-derived Tregs at 1:1 ratio. Cells were cultured (5 × 10^3^ cells/well) in U-bottom 96-well plates with RPMI-1640 medium supplemented with 2-mM L-glutamine, 100 U/ml penicillin, 100 μg streptomycin, and 10% fetal bovine serum (GE Healthcare Life Science, HyClone Laboratories). Cells were stimulated for 5 days in the presence of Dynabeads Human T-Activator CD3/CD28 (Gibco by Life Technologies). Tregs’ suppressive activity was assessed through CFSE-labeled Teffs proliferation by FACS analysis. Furthermore, Tregs were pretreated for 30 min at 37 °C in 5% CO_2_ with 10 µM peptide R29 (Pep R29) [36] or 10 µM AMD3100 (Sigma-Aldrich), CXCR4 antagonist, or 20 µg/mL of human anti-PD-1 (nivolumab), before coculturing with Teffs. Each value obtained from FACS analysis was shown as percentage of coculture Teffs proliferation normalized to Teffs proliferation stimulated alone (set to 100%).

### Cytokine assay

IL-35 and IFNγ were measured by ELISA assay in the culture supernatants. In particular, IL-35 concentration was assessed by Human IL-35 ELISA kit (Boster Biological Technology Co) and IFN-γ was measured by IFN gamma Human ELISA kit (Invitrogen by Thermo Fisher Scientific). Samples were acquired by LB 940 Multimode Reader Mithras (Berthold Technologies).

### RNA extraction, cDNA synthesis and real-time PCR

RNA extraction was performed using TRIzol™ Reagent (Invitrogen, Carsbald, CA, USA), according to the manufacturer’s instructions. RNA was extracted from PB-derived Tregs and Huh7 cell line. Total extracted RNA was used for the reverse transcription reaction performed with SensiFast cDNA Synthesis Kit (Meridian Bioscience, Bioline). Quantitative real-time PCR was performed using SensiMix SYBR Hi-ROX Kit (Meridian Bioscience, Bioline) and data were collected and quantitatively analyzed with 2^−∆Ct^ method on a QuantStudio™ 5 Real-Time PCR System. Primers were designed to assess FOXP3, CXCL12, CXCR4, CCL5, IL-15, CXCL5, Arg-1, N-cad, Vim, CXCL8, TGFβ, and VEGF-A gene expression normalized using beta-2microglobulin (B2M) or actin beta (ACTB) (Table 2). The primer pairs were subjected to a specificity checking process through the Primer3 tool (http://primer3.ut.ee/) publicly available.

**Table 2:** List of primer sequences

Genes	Sequence (5'-3')
FOXP3	FWD: AGCACATTCCCAGAGTTCCT
	REV: TGGCGTAGGTGAAAGGGG
CXCR4	FWD: TGGGTGGTTGTGTTCCAGTTT
	REV: ATGCAATAGCAGGACAGGATGA
CXCL12	FWD: TGTGGCACTCAGATACCGACT
	REV: CCCACAGAGGCCAATCACT
CCL5	FWD: TACACCAGTGGCAAGTGCTC
	REV: TCTCTGGGTTGGCACACAC
CXCL5	FWD: AGACCACGCAAGGAGTTCAT
	REV: TCTTCAGGGAGGCTACCACTT
IL-15	FWD: TCCATCCAGTGCTACTTGTGT
	REV: CTGCACTGAAACAGCCCAAAA
Arg-1	FWD: ACACTCCACTGACAACCACAA
	REV: CTGGCACATCGGGAATCTTTC
N-cad	FWD: CCATCATTGCCATCCTGCTC
	REV: CGGCGTTTCATCCATACCAC
Vim	FWD: GAGAGGAAGCCGAAAACACC
	REV: GCGTTCAAGGTCAAGACGTG
CXCL8	FWD: GACAGCAGAGCACAC
	REV: GGCAAAACTGCACCT
TGFβ	FWD: TCGCCAGAGTGGTTA
	REV: TAGTGAACCCGTTGA
VEGF-A	Hs-VEGF-A-6-SG, QuantiTect Primer Assay (QT01682072)
ACTB	FWD: AGAAAATCTGGCACCACACC
	REV: TAGCACAGCCTGGATAGCAA
B2M	FWD: CATTCCTGAAGCTGACAGCATTC
	REV: TGCTGGATGACGTGAGTAAACC

### Statistical analysis

Data and statistical analyses were performed with GraphPad Prism 8.0.1 (GraphPad Software, Inc., San Diego, CA); data were represented as mean values ± e.s.m. and statistical analyses were carried out using the paired and unpaired two-tailed Student’s *T* test as appropriate. *p* values (p) less than 0.05 were considered statistically significant and reported in figures as follows: * for *p* < 0.05, ** for *p* < 0.01, *** for *p* < 0.001.

## Results

### HCC and CRLM peripheral blood (PB) Tregs are highly represented with HCC Tregs the most active

**Fig. 1:**
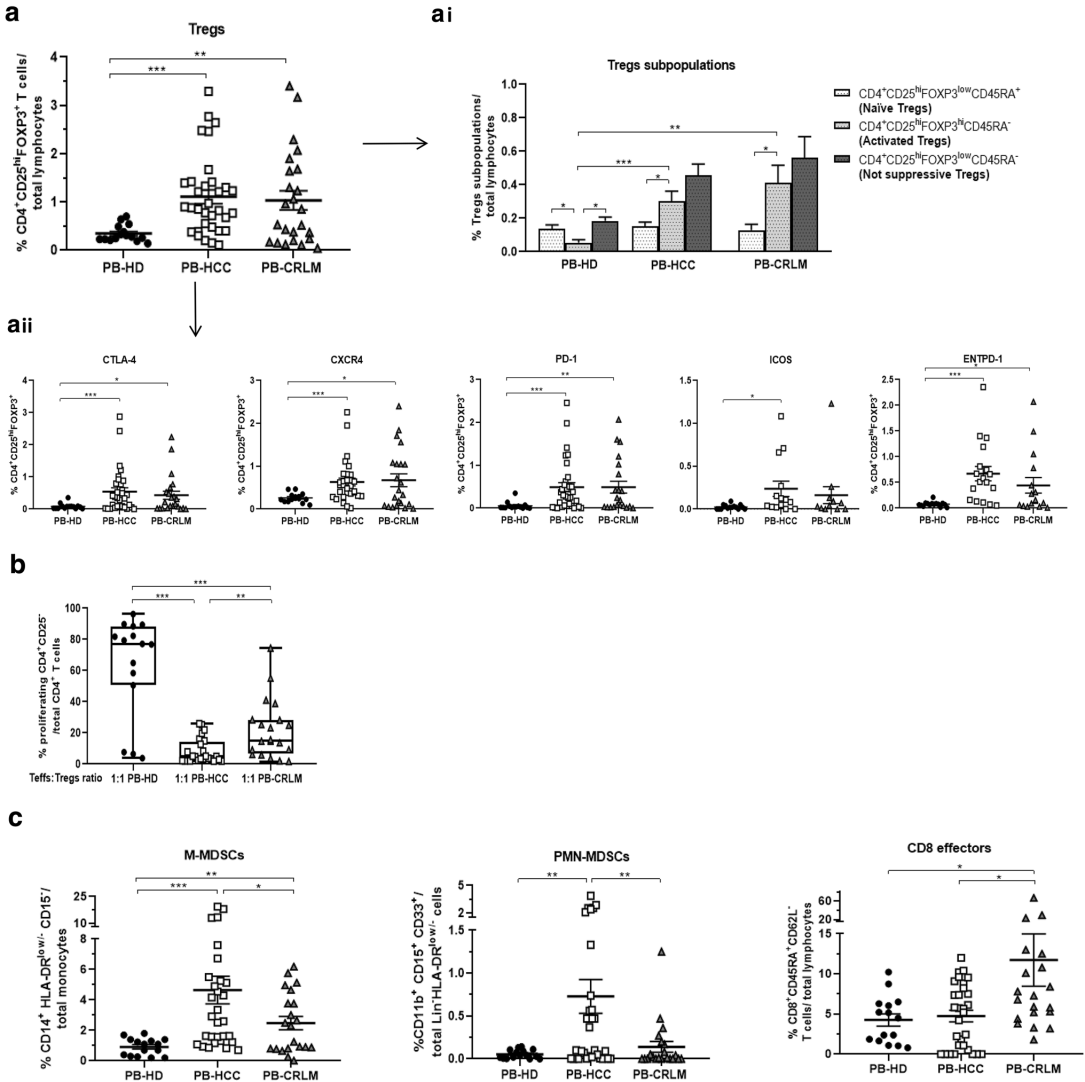
HCC and CRLM peripheral blood (PB) Tregs are highly represented: HCC Tregs are the most active. **a** Phenotypic characterization of peripheral Tregs (CD4^+^CD25^hi^Foxp3^+^ T cells/ total lymphocytes) in 33 HCC and 24 CRLM as compared to 15 HD (HCC vs HD, *p* < 0.001; CRLM vs HD, *p* < 0.01). (**ai**) naïve (CD25^hi^FOXP3^low^CD45RA^+^), activated (CD25^hi^FOXP3^hi^CD45RA^−^), and not suppressive (CD25^hi^FOXP3^low^CD45RA^−^) Tregs in HCC, CRLM, and HD peripheral blood: (activated Tregs: HCC vs HD, *p* < 0.001; CRLM vs HD, *p* < 0.01); (HD: activated vs naïve and not suppressive Tregs, *p* < 0.05); (HCC and CRLM: activated vs naïve Tregs *p* < 0.05). (**aii**) Tregs activation markers in HCC and CRLM compared to HD: CTLA-4, CXCR4, PD-1, and ENTPD1 (HCC vs HD, *p* < 0.001); ICOS (HCC vs HD, *p* < 0.05); CTLA-4, CXCR4, and ENTPD1 (CRLM vs HD, *p* < 0.05); PD1 (CRLM vs HD, *p* < 0.01). **b** Functional PB-derived Tregs from 24 HCC, 20 CRLM patients, and 15 HD by CFSE suppression assay (PB-HCC/CRLM vs PB-HD: *p* < 0.001; PB-HCC vs PB-CRLM: *p* < 0.01). **c** Peripheral M-MDSCs (CD14^+^HLA-DR^low/−^CD15^−^) and PMN-MDSCs (CD11b^+^CD15^+^CD33^+^Lin^−^HLA-DR^low/−^) in HCC and CRLM (M-MDSCs: HCC vs HD: *p* < 0.001; CRLM vs HD: *p* < 0.01; HCC vs CRLM: *p* < 0.05); (PMN-MDSCs: HCC vs HD, *p* < 0.01; HCC vs CRLM, *p* < 0.01). Peripheral CD8^+^ effector cells (CD8^+^CD45R4^+^CD62L.^−^) in HCC and CRLM (CRLM vs HD, *P* < 0.05; HCC vs CRLM, *p* < 0.05)

Tregs, CD4^+^CD25^hi^FOXP3^+^ T cells, were characterized on peripheral blood from 15 HD, 33 HCC, and 24 CRLM patients (Table 1). Higher percentage of Tregs was detected in PB from HCC and CRLM patients as compared to HD (Fig. 1a and Supplementary Fig. 1a and 2a). Tregs were described as naïve (CD4^+^CD25^hi^FOXP3^low^CD45RA^+^), activated (CD4^+^CD25^hi^FOXP3^hi^CD45RA^−^), and not suppressive (CD4^+^CD25^hi^FOXP3^low^CD45RA-) [37]. Activated Tregs were significantly higher in PB from HCC and CRLM as compared to HD (Fig. 1ai and Supplementary Fig. 3). Moreover, HCC and CRLM Tregs expressed significantly high CTLA4, CXCR4, PD-1, and ENTPD-1 (HCC vs HD, p < 0.001), while only Tregs from HCC significantly overexpressed ICOS (HCC vs HD, p < 0.05) (Fig. 1aii and Supplementary Fig. 1b and 2B). To verify whether the activated phenotype corresponds to a suppressive function, Tregs-dependent Teffs proliferation was evaluated. Tregs from peripheral blood of HCC and CRLM patients were more suppressive than HD-derived Tregs. Interestingly, PB-HCC Tregs more potently suppress Teffs proliferation as compared to PB-CRLM at 1:1 Teffs/Tregs ratio and in a dose escalation experiments (Fig. 1b and Supplementary Fig. 4 and 5). Accordingly, in supernatants of Teffs:Tregs coculture, IL-35 increased from PB-HCC and PB-CRLM as compared to HD (Supplementary Fig. 6a, b), and IFNγ was reduced in PB-HCC and PB-CRLM as compared to Teff alone (Supplementary Fig. 7). Monocytic (M) and Polymorphonuclear (PMN) MDSCs were also evaluated in peripheral blood. In HCC and CRLM higher percent of (M)-MDSCs (CD14^+^HLA^−^DR^low/−^CD15^−^) was observed as compared to HD, while only in HCC (PMN)-MDSCs (CD11b^+^CD15^+^CD33^+^Lin^−^HLA-DR^low/−^) were highly represented. Interestingly, M-MDSCs and PMN-MDSCs were significantly higher in PB-HCC as compared to PB-CRLM, in accordance with low CD8^+^ effector cells (CD8^+^CD45R4^+^CD62L^−^) in PB-HCC (Fig. 1c). Thus, higher percentage of functional Tregs and MDSCs was detected in peripheral blood of both HCC and CRLM patients, but Tregs from HCC patients were more immunosuppressive as compared to CRLM Tregs.

### Tregs are highly represented in HCC and CRLM tumor tissue (TT) as compared to peritumoral tissue (PT): ENTPD-1^+^Tregs and MDSCs characterized a more immunosuppressive TME in HCC

**Fig. 2:**
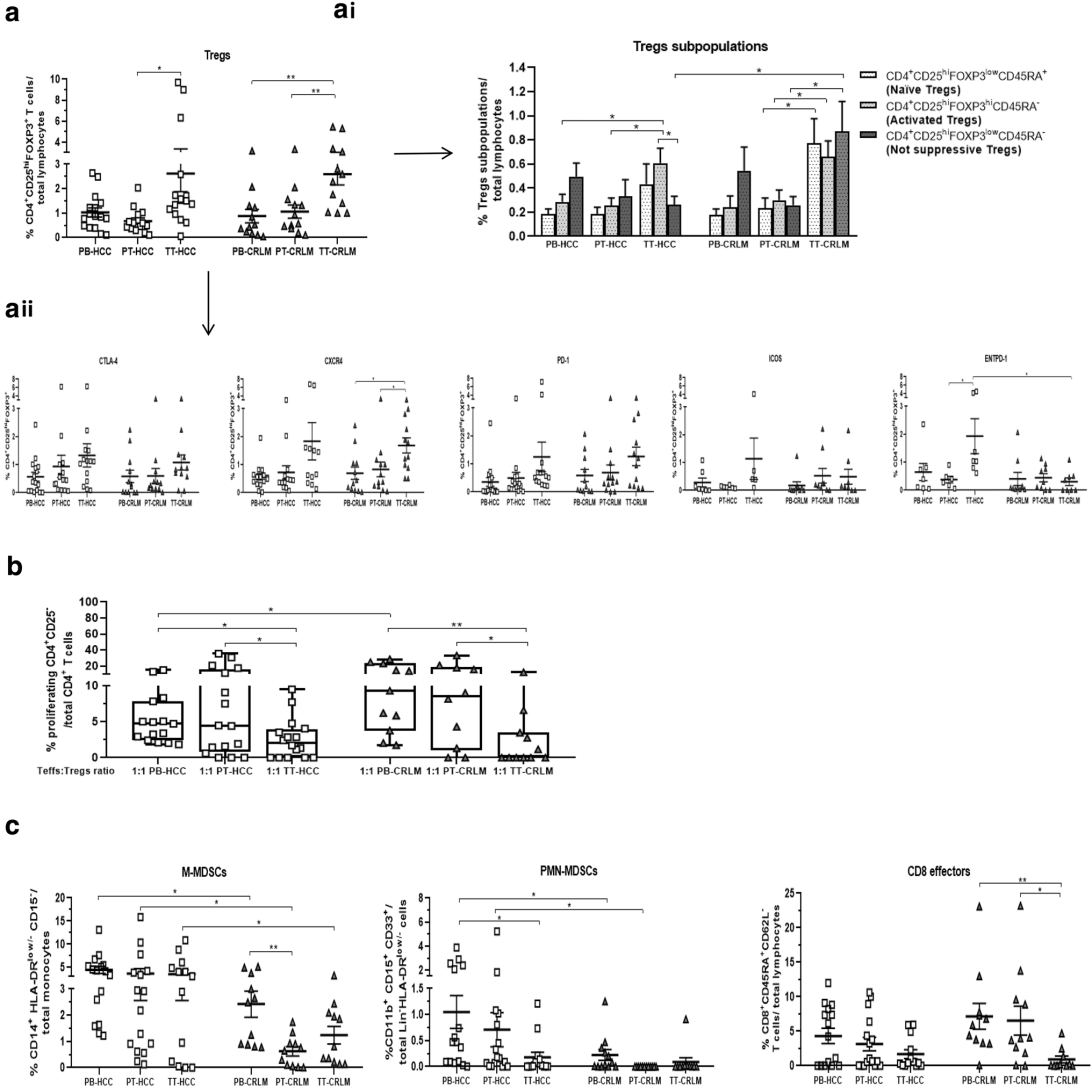
Tregs are highly represented in HCC and CRLM tumor tissue (TT) as compared to peritumoral tissue (PT). ENTPD-1^+^Tregs and MDSCs characterized a more immunosuppressive TME in HCC. **a** Paired peripheral, peritumoral and tumor Tregs by flow cytometry in 16 HCC and 12 CRLM patients (TT-HCC vs PT-HCC, *p* < 0.05; TT-CRLM vs PT-CRLM: *p* < 0.01). (**ai**) Naïve, activated and not suppressive Tregs in HCC and CRLM patients: (activated Tregs: TT-HCC/CRLM vs PT-HCC/CRLM, *p* < 0.05; naïve and not suppressive: TT-CRLM vs PT-CRLM, *p* < 0.05); not suppressive Tregs (TT-HCC vs TT-CRLM: *p* < 0.05); (TT-HCC: activated Tregs vs not suppressive, *p* < 0.05). (**aii**) Tregs’ activation markers in HCC and CRLM patients: CXCR4 (TT-CRLM vs PB-/PT-CRLM: *p* < 0.05); ENTPD1 (TT-HCC vs PT-HCC: *p* < 0.05); (TT-HCC vs TT-CRLM: *p* < 0.05). **b** Functional characterization of PB-, PT-, and TT- derived Tregs from HCC and CRLM patients: (TT-HCC/CRLM vs PT-HCC/CRLM: *p* < 0.05); (TT-HCC/CRLM vs PB-HCC/CRLM: *p* < 0.01 and *p* < 0.05); (PB-HCC vs PB-CRLM: *p* < 0.05). **c** M-MDSCs and PMN-MDSCs in HCC and CRLM: (M-MDSCs: PB-/PT-/TT-HCC vs PB-PT-TT-CRLM: *p* < 0.05); (PMN-MDSCs:PB-/PT-HCC vs PB-/PT-CRLM: *p* < 0.05). CD8.^+^ effector cells in HCC and CRLM (TT-CRLM vs PT-CRLM: *p* < 0.05; TT-CRLM vs PB-CRLM: *p* < 0.01)

At time of surgery, we collected 16 HCC and 12 CRLM with paired PB, PT, and TT specimens evaluated for phenotypic and functional analysis. A higher percentage of Tregs was revealed in tumor as compared to peritumoral tissues in both tumors (Fig. 2a and Supplementary Fig. 1a and 2a). In TT-CRLM, naïve, activated and not suppressive Tregs were significantly higher, while in TT-HCC only activated Tregs subpopulation increased as compared to PT; moreover, in TT-HCC a significantly lower percentage of not suppressive Tregs was observed as compared to TT-CRLM (Fig. 2ai and Supplementary Fig. 3). CXCR4 was overexpressed in Tregs from TT-CRLM as compared to PT; interestingly, ENTPD1 was significantly overexpressed in TT-HCC as compared to TT-CRLM (p < 0.05) (Fig. 2aii, Supplementary Fig. 1b and 2b). Functional analysis revealed that Tregs isolated from tumor tissue more potently suppress Teffs proliferation as compared to PB- and PT-derived Tregs in both HCC and CRLM tumors (Fig. 2b and Supplementary Fig. 5) as confirmed with IL-35 (Supplementary Fig. 6c). Furthermore, higher percentage of M-MDSCs and PMN-MDSCs was observed in HCC as compared to CRLM with slightly lower CD8^+^ effectors in PT-HCC (Fig. 2c). To indirectly evaluate the effect of TME on proliferation, the percent of Ki67-positive cells was considered. A higher percentage of Ki67 expressing cells was detected in human hepatoma cells cocultured with HCC-PBMC compared to HD-PBMC (Supplementary Fig. 8). Moreover, HCC/CRLM tumor dimension correlated with the percentage of PT and TT Tregs and PT- and TT-MDSC (Supplementary Fig. 9a-b). Thus, functional Tregs accumulate at the tumor site in both tumors, but in HCC Tregs displayed a stronger immunosuppressive phenotype as suggested by higher expression of ENTPD1. In addition, higher M- and PMN-MDSCs also contribute to immunosuppressive TME in HCC as compared to CRLM.

### Ex vivo CXCR4 antagonism impairs the suppressive capability of PB-HCC/CRLM Tregs

**Fig. 3:**
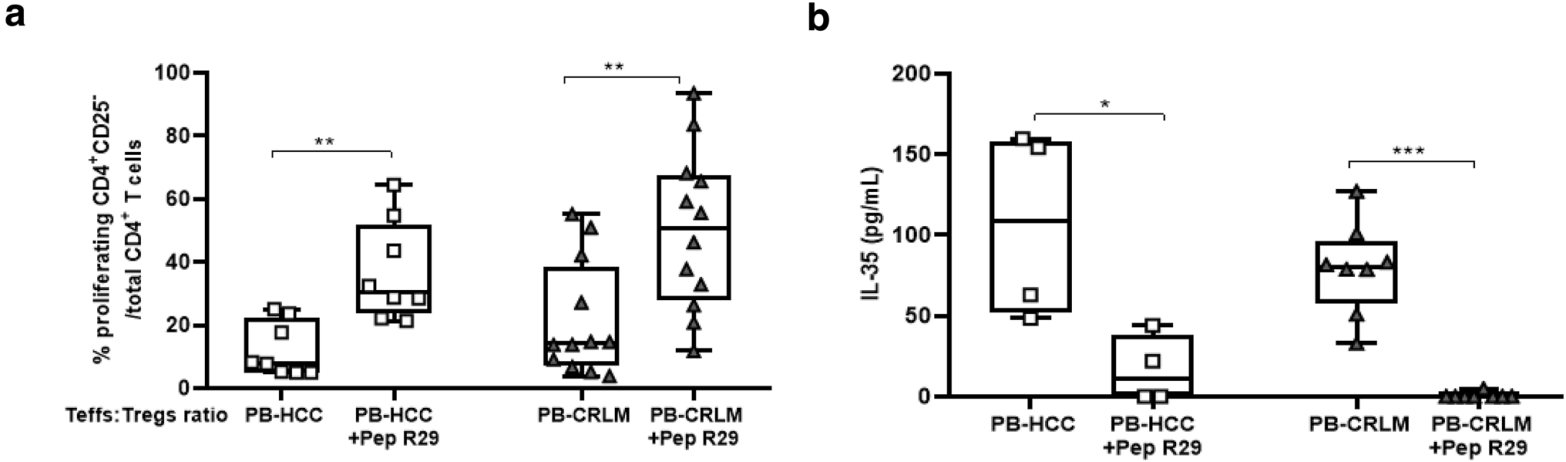
Ex vivo CXCR4 antagonism impairs PB-HCC/CRLM Tregs suppressive capability. **a** Functional PB-derived Tregs from 8 HCC and 12 CRLM treated for 30 min at 37 ℃ in 5% CO_2_ with Pep R29 (10 μM) and then added at 1:1 ratio to Teffs for 5 days (HCC: 1:1 vs 1:1 + Pep R29, *p* < 0.01;) (CRLM: 1:1 vs 1:1 + Pep R29, *p* < 0.01). **b** IL-35 concentration (pg/ml) in supernatant of CFSE assay from Tregs pretreated with Pep R29 (10 μM) in 4 HCC and 8 CRLM patients by ELISA: (HCC: 1:1 vs 1:1 + Pep R29, *p* < 0.05) (CRLM: 1:1 vs 1:1 + Pep R29, *p* < 0.001)

As Tregs overexpressed CXCR4 in HCC and CRLM patients, the effect of CXCR4 blockade was evaluated through the CXCR4 inhibitor peptide R29. An efficient reversal of the suppressive capability of Tregs was detected in both tumors in the presence of Pep R29 (Fig. 3a), AMD3100, and anti-PD1 (Supplementary Fig. 10a-b). As previously shown, Pep R29 did not affect the proliferation of Teffs in the presence of HD-derived peripheral Tregs [31]. To confirm the effect of CXCR4 antagonism on the function of Tregs, Tregs-secreted IL-35 was evaluated. In Fig. 3b, a significant decrease of IL-35 secretion was detected in Pep R29-treated PB-HCC/CRLM-derived Tregs. In addition, Tregs treatment with AMD3100 and anti-PD1 reduced IL-35 in PB-Tregs from both patients (Supplementary Fig. 10c). Moreover, Pep R29 treatment reduced Foxp3 expression in peripheral blood Tregs from HCC and CRLM patients (Supplementary Fig. 10d). Overall, these data suggest that CXCR4 antagonism may impair Tregs’ function in liver cancer patients.

### HCC and CRLM intrinsic tumor features

**Fig. 4:**
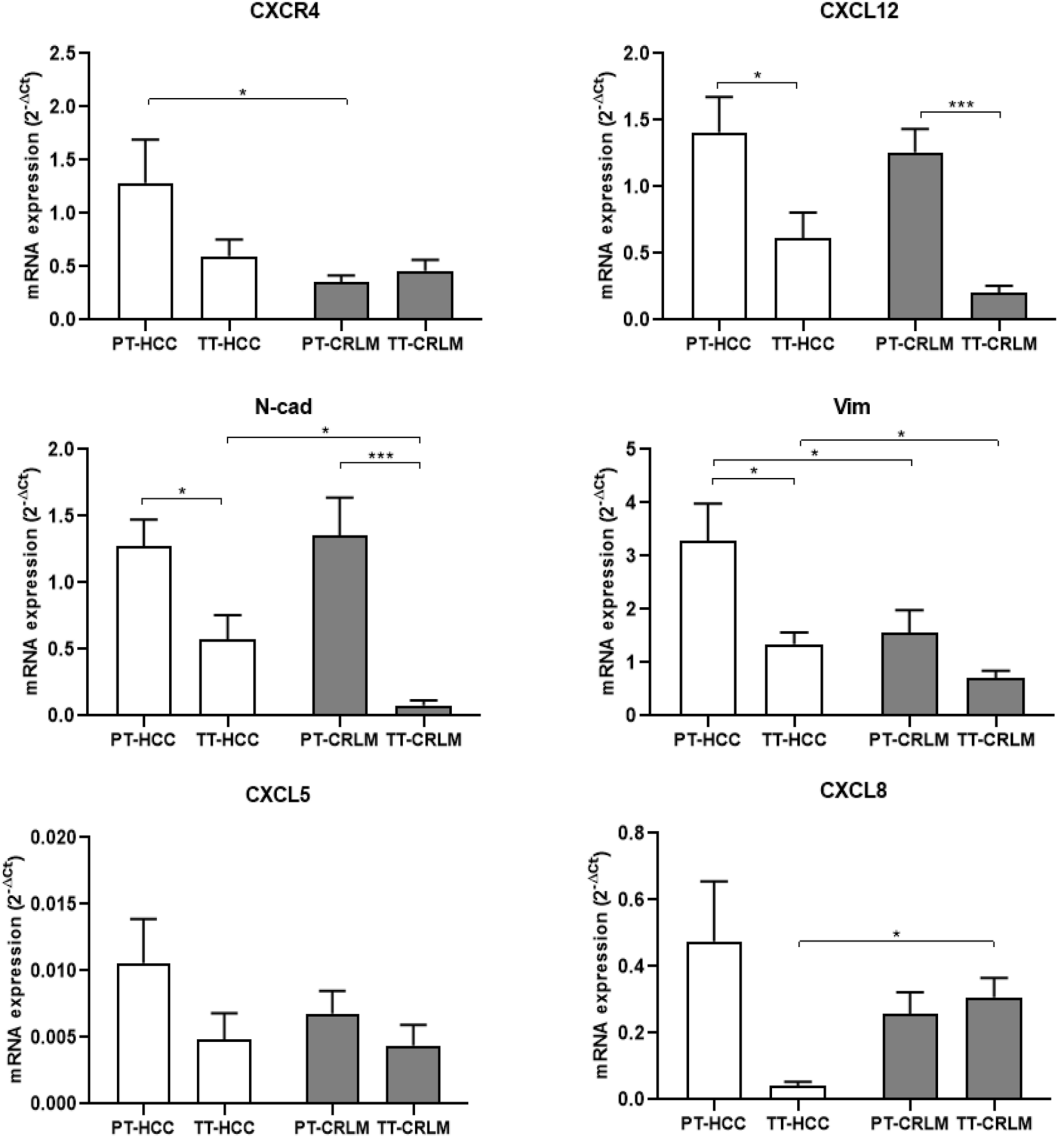
CXCR4 and mesenchymal markers defined HCC. Real-time PCR was performed to define CXCR4, CXCL12, N-cad, Vim, CXCL5 and CXCL8 expression in tumor and peritumoral tissue of HCC and CRLM patients. ACTB was used for normalization of target gene expression. CXCR4 (19 PT-HCC vs 23 PT-CRLM, *P* < 0.05); CXCL12 (20 HCC: PT vs TT, *p* < 0.05; 18 CRLM: PT vs TT, *p* < 0.001); N-cad (17 HCC: PT vs TT, *p* < 0.05; 21 CRLM: PT vs TT, *p* < 0.001) (17 TT-HCC vs 21 TT-CRLM, *p* < 0.05); Vim (21 HCC: PT vs TT, *p* < 0.05) (21 PT-HCC vs 22 PT-CRLM, *p* < 0.05) (21 TT-HCC vs 22 TT-CRLM, *p* < 0.05); CXCL8 (5 TT-HCC vs 10 TT-CRLM, *p* < 0.05)

To define HCC- or CRLM-intrinsic tumor features on liver TME, CXCR4, CXCL12, N-cadherin (N-cad), vimentin (Vim), CXCL5, and CXCL8 expression was evaluated in HCC and CRLM tissues. As shown in Fig. 4, CXCR4 expression was comparable between TT in HCC and CRLM, but significantly lower in PT-CRLM, while CXCL12 was significantly lower in TT-HCC/CRLM as compared to PT tissue. N-cadherin was overexpressed in PT compared to TT in both tumors, while Vim was overexpressed in PT compared to TT only in HCC tissue; interestingly, both N-cad and Vim were higher in TT-HCC as compared to TT-CRLM, defining a mesenchymal transition in HCC cells. No significant change in CXCL5 expression was observed in both tumors, instead CXCL8 was lower in TT-HCC as compared to TT-CRLM.

### Intrinsic tumor features modifying tumor microenvironment

**Fig. 5:**
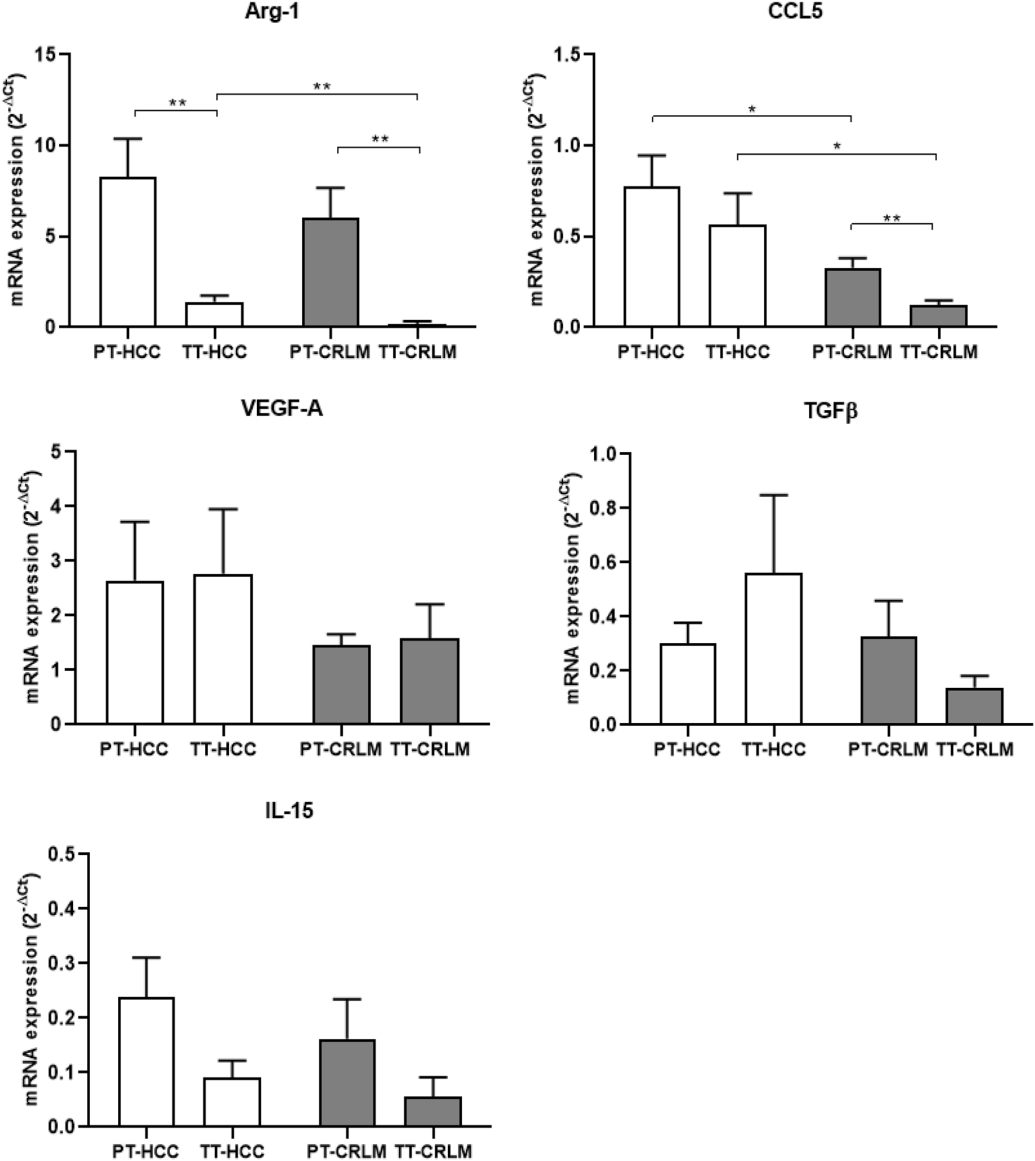
Intrinsic tumor features modifying the tumor microenvironment. HCC-TME overexpressed ARG-1 and CCL5. Real-time PCR was performed to define Arg-1, CCL5, VEGF-A, TGFβ, and IL-15 expression in tumor and peritumoral tissue of HCC and CRLM patients. ACTB was used as normalization of target gene expression. Arg-1 (20 HCC: PT vs TT P < 0.01) (22 CRLM: PT vs TT, *p* < 0.01) (20 TT-HCC vs 22 TT-CRLM, *p* < 0.01); CCL5 (20 PT-HCC vs 22 PT-CRLM, *p* < 0.05) (22 CRLM: PT vs TT, *p* < 0.01) (20 TT-HCC vs 22 TT-CRLM *p* < 0.05)

**Fig. 6:**
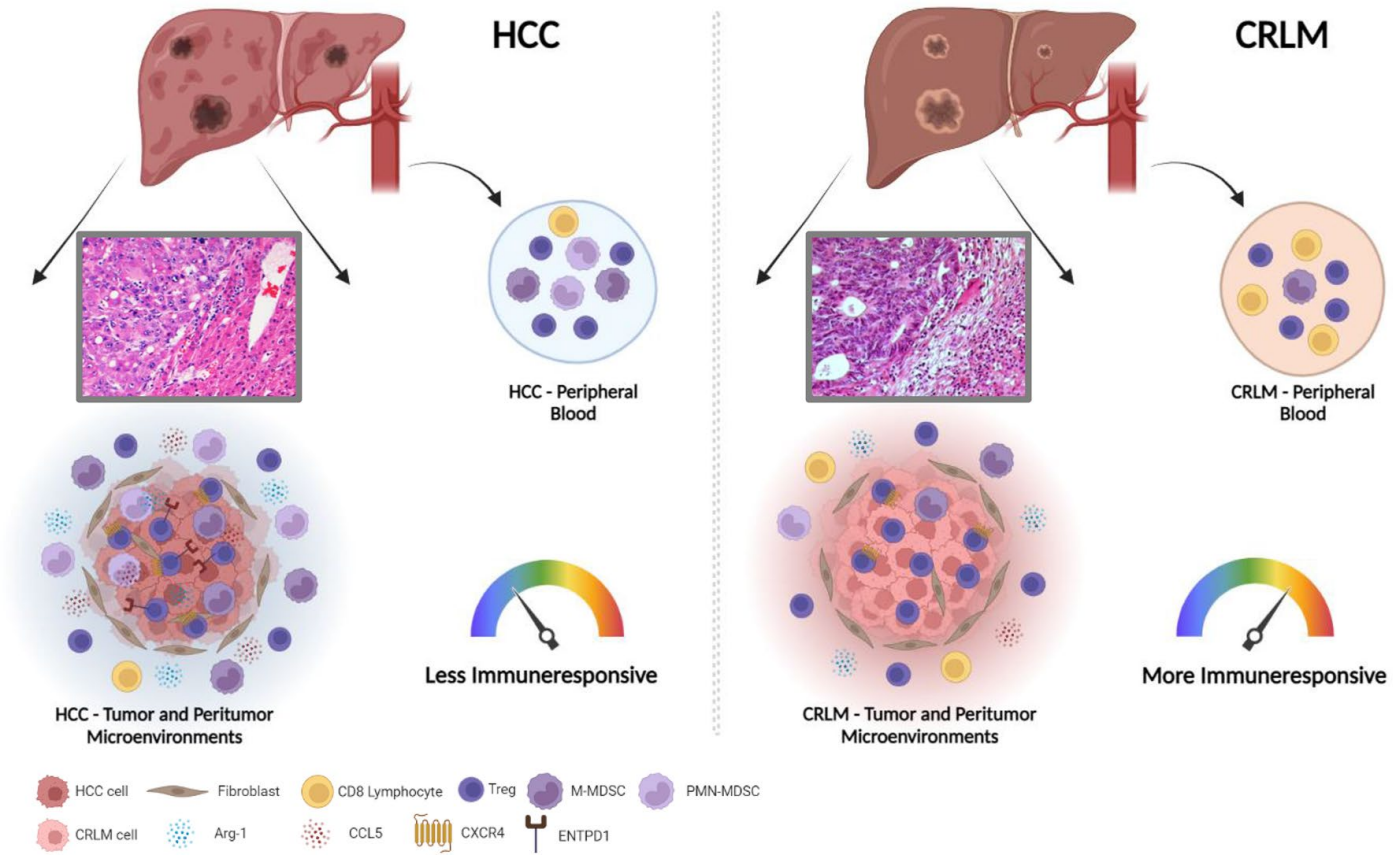
Proposed landscape of the tumor microenvironment in HCC and CRLM

To characterize HCC- or CRLM-specific TME component, the expression of arginase-1 (Arg-1), CCL5, VEGF-A, TGFβ, and IL-15 was evaluated. In Fig. 5, higher Arg-1 was detected in PT as compared to TT tissue in both tumors. Interestingly, increased Arg-1 expression was observed in TT-HCC as compared to TT-CRLM, in accordance with higher prevalence of MDSCs and mesenchymal markers in HCC. Moreover, higher CCL5 expression was detected in PT-CRLM as compared to TT and interestingly higher CCL5 expression in TT-HCC as compared to TT-CRLM. Analysis of VEGF-A and TGFβ expression showed no significant changes, even if VEGF-A appeared to be higher in HCC as compared to CRLM, and the highest TGFβ was observed in TT-HCC. No changes in IL15 expression was reported in HCC and CRLM samples. Thus, HCC is characterized by higher arginase-1 and CCL5 as compared to CRLM tissues, suggesting that HCC-TME is oriented toward a stronger immunosuppression. In Fig. 6 the described results are recapitulated.

## Discussion

In the present study, peripheral, peritumoral, and tumoral Tregs were analyzed in HCC and CRLM to evaluate the role of TME on the development of primary and secondary lesions. As previously reported, higher percentage of Tregs was detected in the peripheral blood of HCC and CRLM patients [38, 39]. Suppressive Tregs are characterized by high expression of CTLA-4, HLA-DR, and ICOS; poor production of IL-2 or IFN-γ; high demethylation rate of FOXP3 and strong suppression of responder cells proliferation in vitro [40]. Herein, HCC and CRLM patients’ peripheral Tregs displayed a robust activated phenotype CTLA4^+^, CXCR4^+^, PD-1^+^, and ENTPD-1^+^, while higher ICOS^+^ Tregs, potent and suppressive in immune escape, [41] were detected only in HCC patients. At the tumor site, higher percentage of Tregs was reported in both HCC and CRLM, as shown by previous evidence [42]. Notably, not suppressive Tregs, characterized by low demethylation rate of FOXP3 and unable to suppress T-effector proliferation [40], were reduced in HCC as compared to CRLM. ENTPD1^+^Tregs are regulators of suppressive function; in HCC they added prognostic power to FOXP3^+^Tregs, as ENTPD1^+^Tregs independently predicts poor outcome after radical resection [43]. We reported higher ENTPD1^+^Tregs in TT-HCC as compared to TT-CRLM. CD39 is mainly a (FOXP3 +) Treg marker [44]; in fact in melanoma and colon cancer models, ENTPD1-deficient mice displayed impaired Treg-suppressive functions [45]. In HCC and in colon cancer patients, CD39 overexpression in Tregs has a poor prognostic value [46, 47]. Currently, there are three agents targeting CD39 in human studies: SRF617, a monoclonal antibody that binds and inhibits CD39; TTX-030 (Tizona Therapeutics), an huIgG4 enzymatic inhibitor in combination with pembrolizumab, doxetaxel, gemcitabine, nab-paclitaxel; IPH5201 (Innate Pharma/AstraZeneca) antibody, an huIgG1 in combination with durvalumab with or without oleclumab [48]. Herein, in HCC higher percentage of peripheral M- and PMN-MDSCs was reported as compared to CRLM, supported by a concomitant reduction of CD8^+^ effector cells. Higher M-MDSCs at the tumor and peritumoral site and higher PMN-MDSCs at the peritumoral site were detected in HCC as compared to CRLM, supported by a slight decrease of CD8^+^ effector cells at the HCC peritumoral site. In the context of the immune microenvironment, neutrophils have emerged as a unique component of the inflammatory response that facilitates intercellular cross talk between tumor cells and the TME [49].

Altogether, these data suggest that HCC is characterized by a prevalence of tumor-activated Tregs and low not suppressive Tregs subpopulation, high ENTPD1^+^Tregs and MDSCs infiltration, and designing a more immunosuppressive TME in HCC as compared to CRLM. In a meta-analysis, it was reported that high tumor and peripheral Tregs associated with better OS and DFS in HCC [50]. This finding is relevant, as Tregs are detected in peripheral blood and allow real-time monitoring compared with Tregs in the tumor. HCC can recruit Tregs in peripheral blood and convert CD4^low^CD25^low^to CD4^high^CD25^high.^ Besides, tumor immune escape occurs not only in local, but also in systemic immunity. Our functional data suggest that in HCC, Tregs are more suppressive than in CRLM and express a high level of CXCR4. Previous evidence demonstrates that CXCR4 inhibition impairs the function of Tregs in renal cancer and malignant mesothelioma [31, 51]. Herein, HCC- and CRLM-PB-Tregs overexpress CXCR4 concomitantly with CTLA4 and PD-1. Highly selective small molecule CXCR4 antagonist suppresses tumor growth and prevents distant metastasis and TAMs infiltration in HCC [28], while the canonical CXCR4 inhibitor, AMD3100, reduces MDSCs-mediated murine HCC cell migration to the spleen and liver [52]. A newly developed CXCR4 antagonist, peptide R29, reverts peripheral Tregs’ suppressive capability in primary renal cancer patients [31]. Herein, CXCR4 blocking by peptide R29 impairs the suppressive function of Tregs in both HCC and CRLM patients, suggesting that CXCR4 antagonism is a possible strategy for liver cancer patients. CXCR4 and mesenchymal markers were evaluated in HCC and CRLM tissues. CXCR4 is expressed in liver cells (HSCs and LSECs) and in cancer cells [53] where correlates to HCC worse prognosis. In HCC, the upregulation of N‐cadherin correlated with post-operative recurrence. Vimentin, EMT reprogramming protein, confers migratory and invasive phenotypes [54] and is highly expressed in hepatic cancers [55]. CXCL5, the ligand of CXCR2, is derived from primary tumor cells, but it is also secreted by immune cells in the TME [56]. CXCL8 and its receptors (CXCR1, CXCR2, and Duffy antigen receptor for chemokines (DARC)) are associated with the development of colorectal cancer and its liver metastases. In our experience, CXCR4, N-cad and Vim were higher in HCC as compared to CRLM defining mesenchymal transition in HCC tumors. Arginase-1, produced by myeloid cells, impairs antitumor response [57], and CCL5, mainly expressed by T lymphocytes, macrophages, platelets, tumor cells [58] and MDSCs, resulted in a direct CCR5-dependent recruitment of Treg cells in TME [59]. In HCC, Arg-1 induces N-cad and Vim, which promote EMT [60]. In CRLM, T cell-CCL5 localized mainly in peritumoral stroma and stimulates protumoral effects via CCR5 [61]. In the study, higher expression of Arg-1 was reported at the peritumoral site in both tumors and higher expression of CCL5 at the peritumoral site in CRLM; notably, the increased immunosuppressive Arg-1 and CCL5 in HCC compared to CRLM emphasizes the existence of an HCC-TME strongly oriented toward immunosuppression. In conclusion, our data demonstrate that both tumors contain high number of Tregs, though HCC-derived Tregs displayed more active phenotype and suppressive function. A more suppressive TME in HCC derives from high immunosuppressive molecules, such as Arg-1 and CCL5, induced by a mesenchymal environment. CXCR4 inhibition is able to prevent T cell resistance and may therefore be a target for immunotherapeutic intervention in liver cancer patients.

### Supplementary Information

Below is the link to the electronic supplementary material.

Supplementary file1 (PDF 792 KB)

Supplementary file2 (DOCX 46 KB)

## Data Availability

Data are available on reasonable request.
