# Supplementary material for: Hepatocellular carcinoma (HCC) tumor microenvironment is more suppressive than colorectal cancer liver metastasis (CRLM) tumor microenvironment
Source: Hepatol Int. 2023 May 4;18(2):568–81. doi: 10.1007/s12072-023-10537-6 (PMC11014815; doi:10.1007/s12072-023-10537-6)

Supplementary Figure 1. Representative plot of Treg cells from PB-HD and PB-HCC, PT-HCC and TT-HCC patient

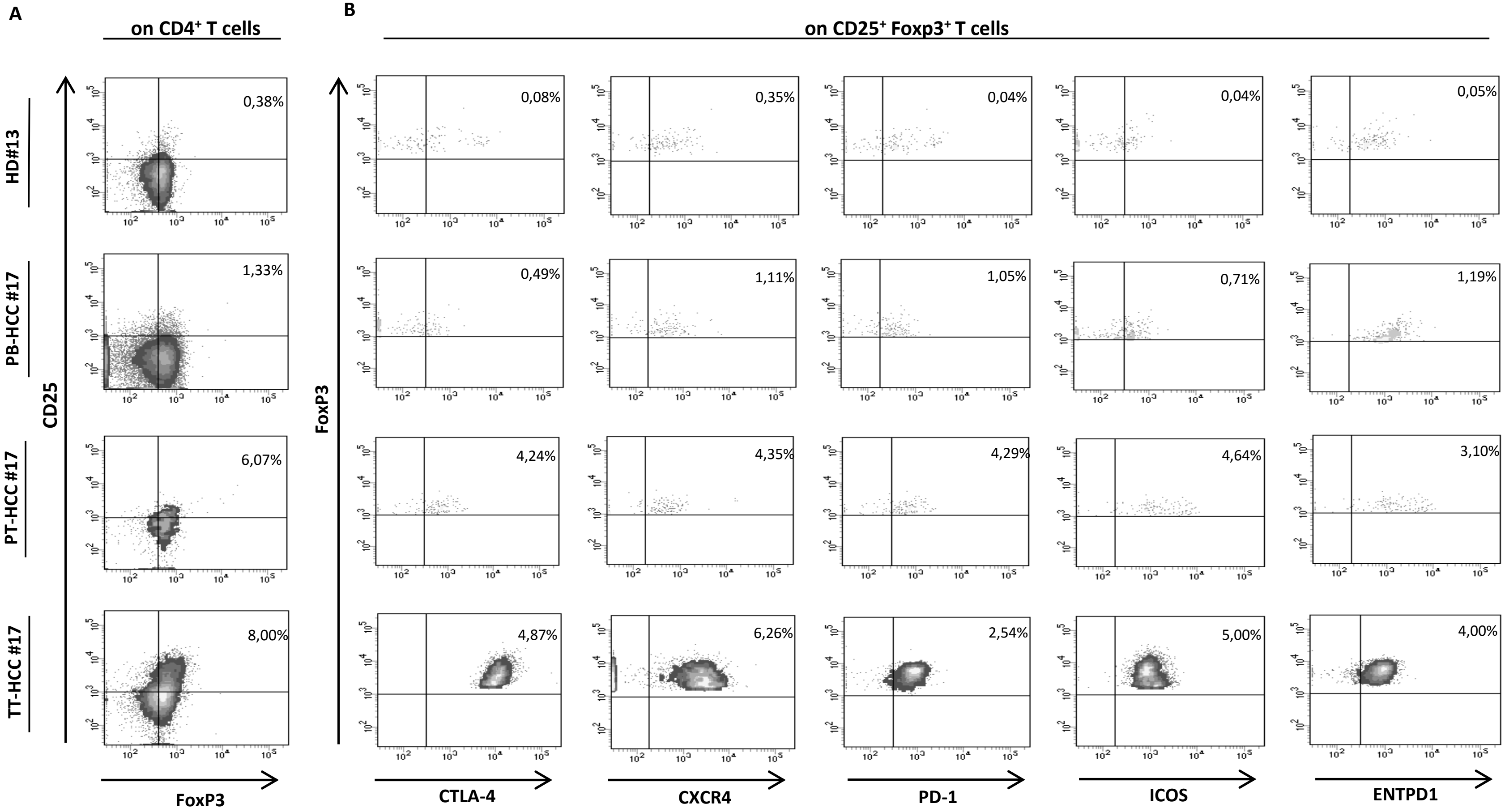

Supplementary Figure 2. Representative plot of Treg cells from PB-HD and PB-CRLM, PT-CRLM and TT-CRLM patient

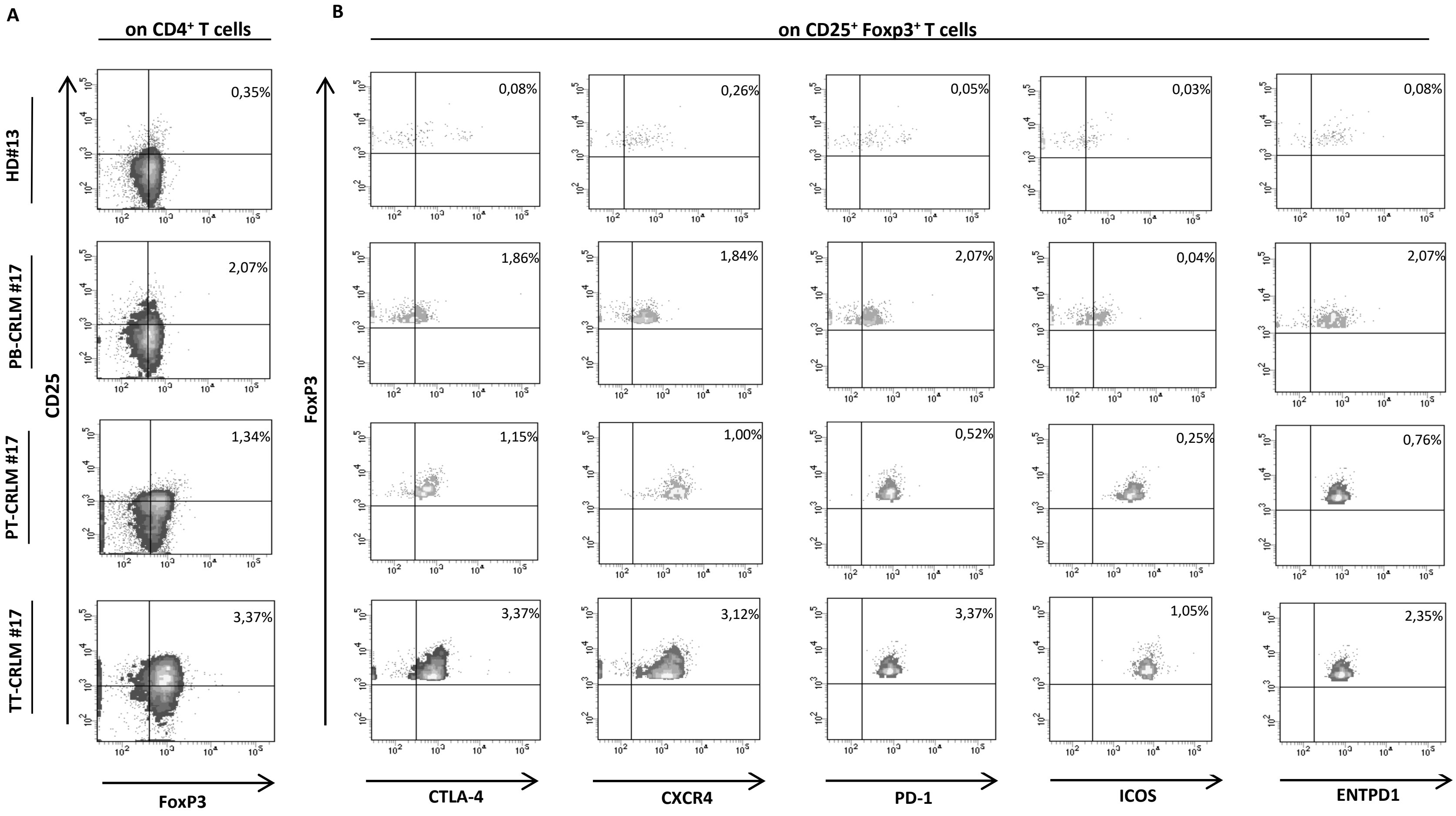

Supplementary Figure 3. Representative plot of Naïve, Activated and Not suppressive Treg cells from HCC and CRLM

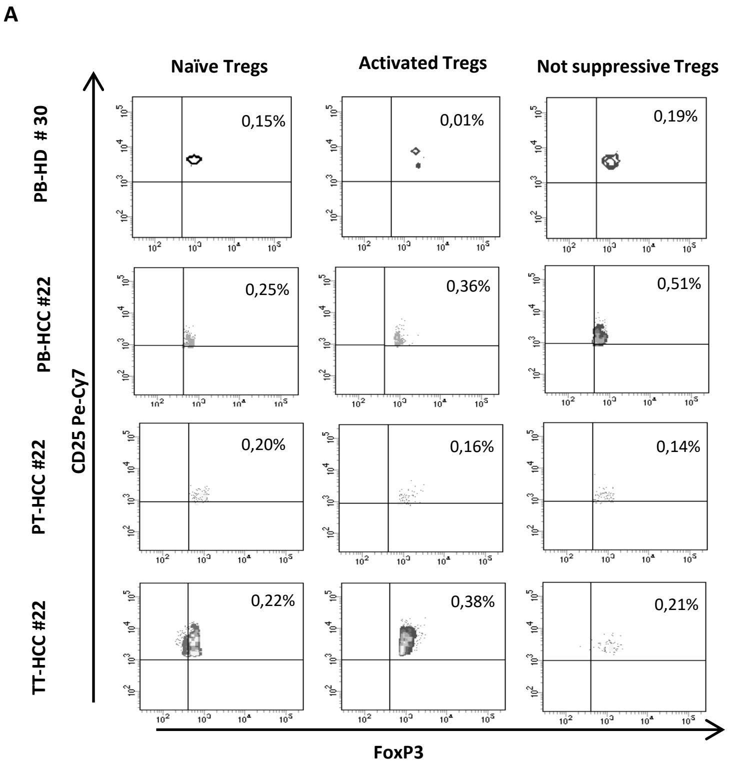

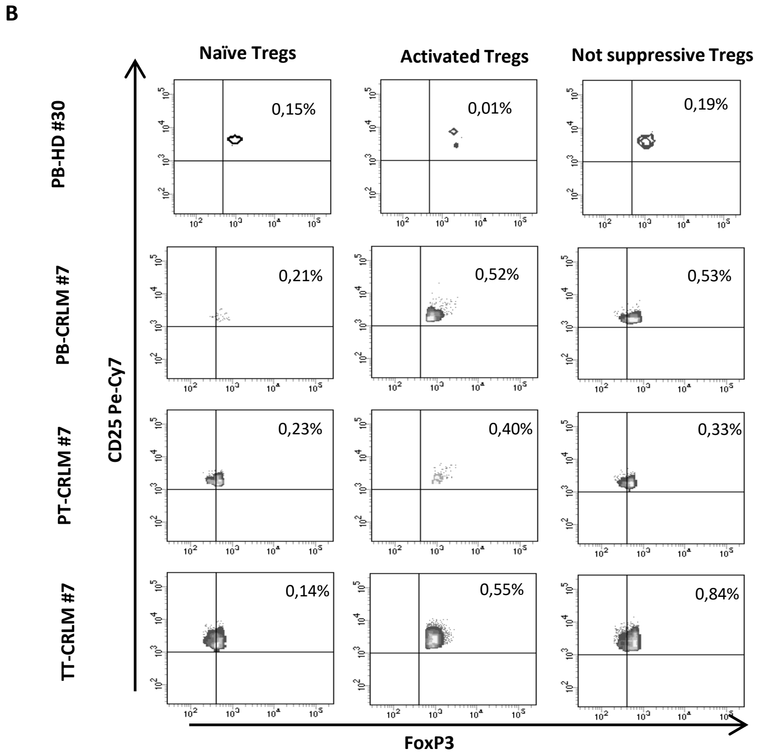

Supplementary Figure 4. Dose escalation functional assay of Tregs suppressive activity in PB of HD subjects, HCC and CRLM patients.

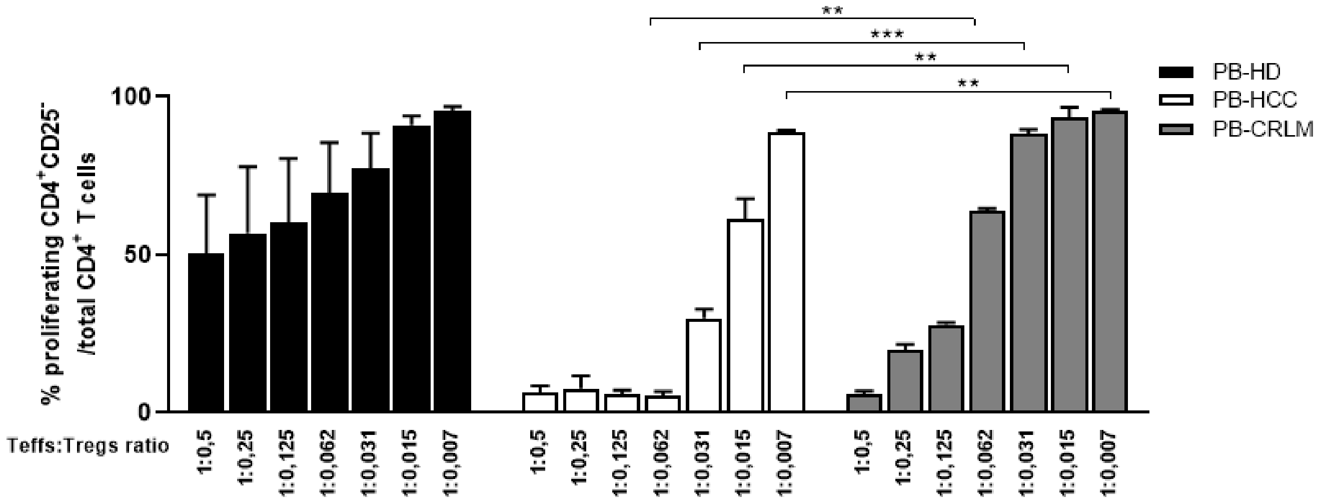

Supplementary Figure 5. Representative functional analysis of Tregs suppressive activity in PB/PT/TT of HCC and CRLM patients.

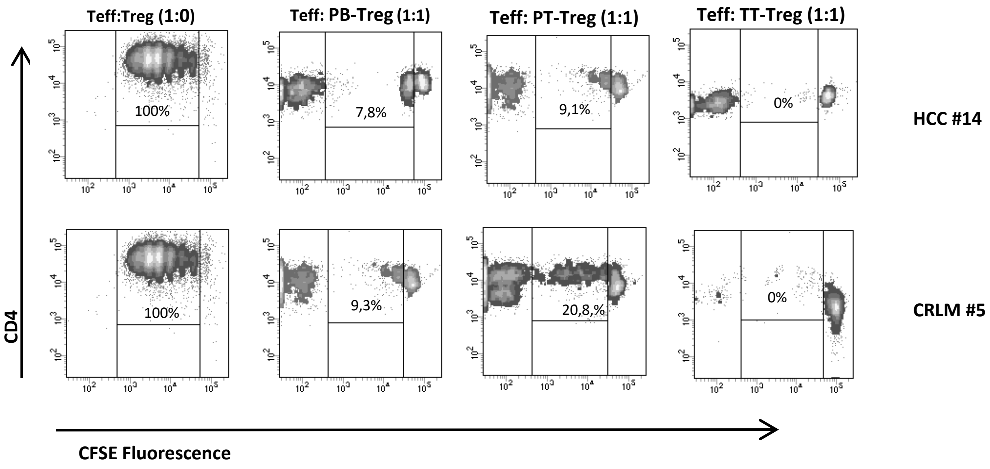

Supplementary Figure 6. IL-35 secretion by Tregs and Teffs of PB-HD, -HCC and –CRLM

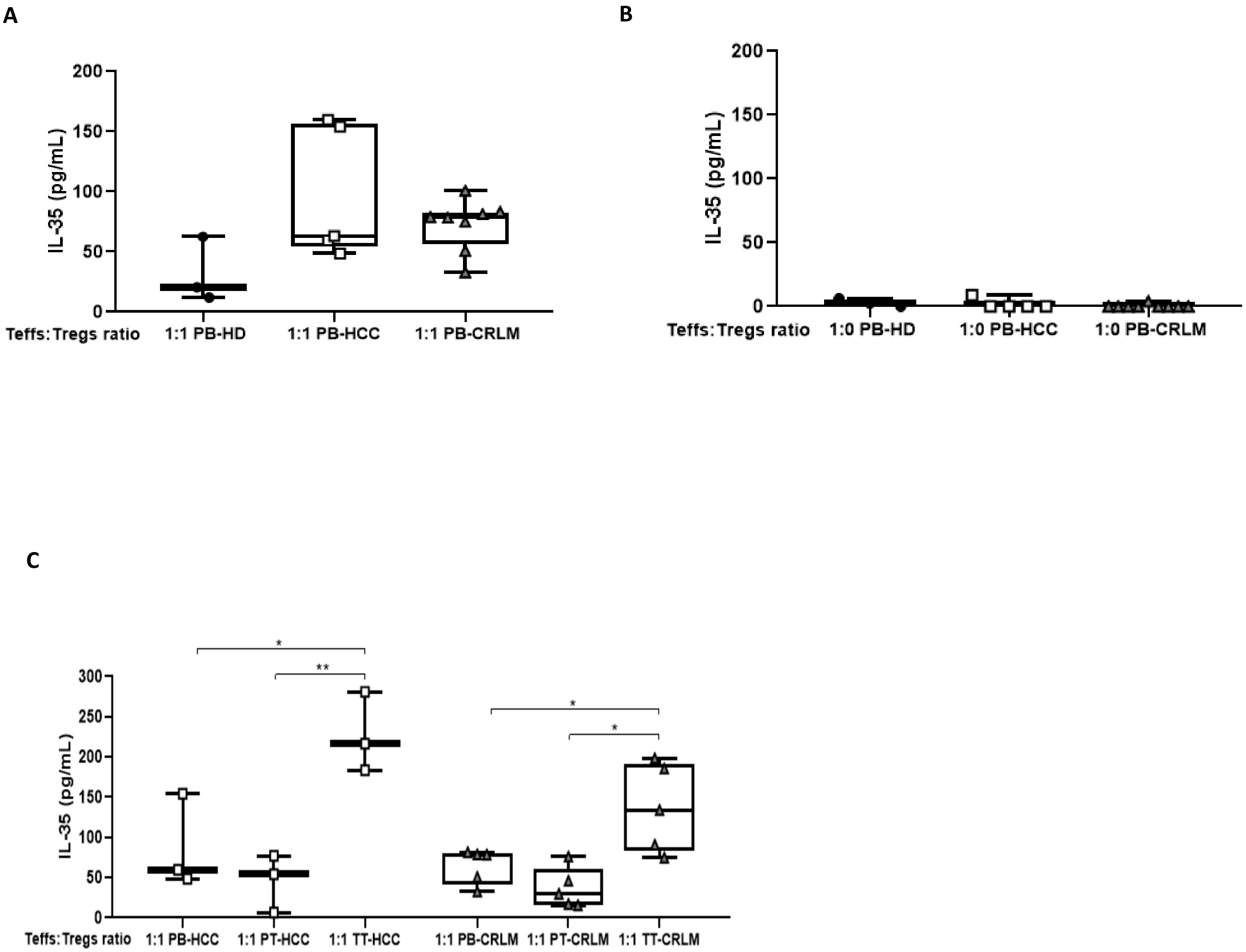

Supplementary Figure 7. Proliferation and IFN-γ secretion in Teffs of HCC and CRLM

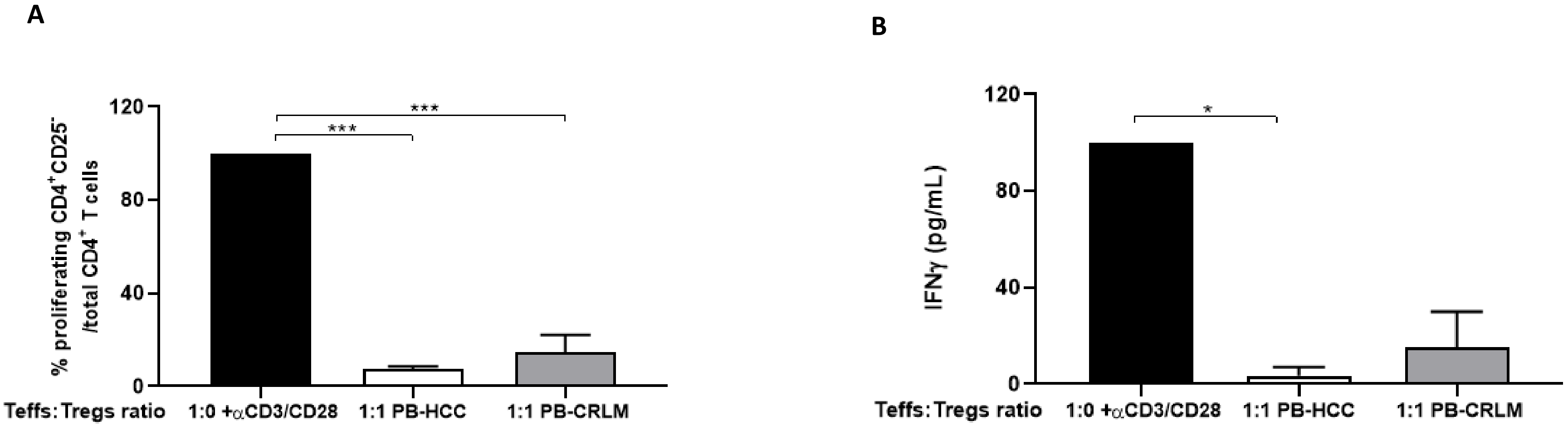

Supplementary Figure 8. Ki67 frequence of positive cells in coculture of tumor cells and PBMC from HCC and CRLM patients

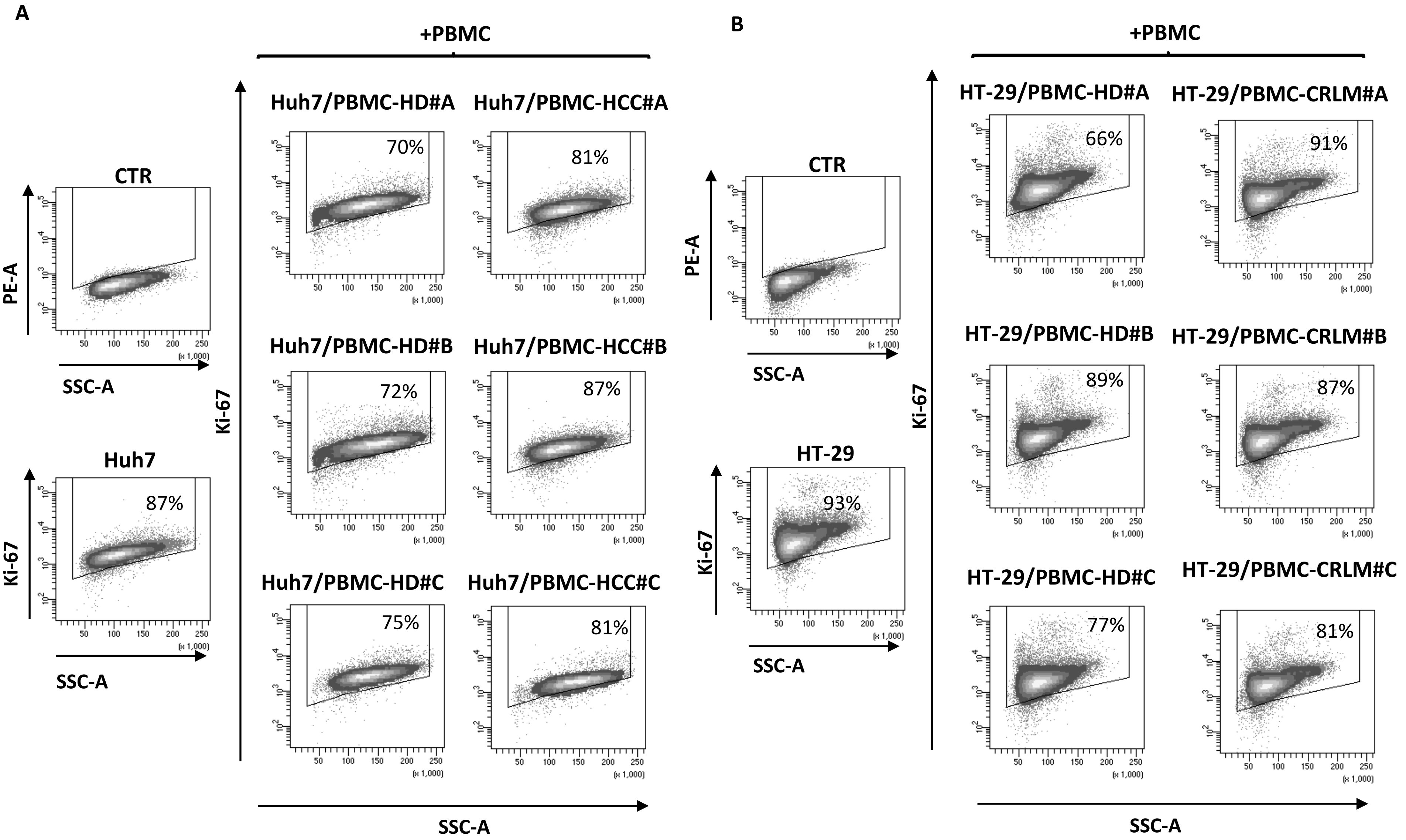

Supplementary Figure 9. Higher percentage of PT-TT-Tregs and MDSCs in HCC tumors

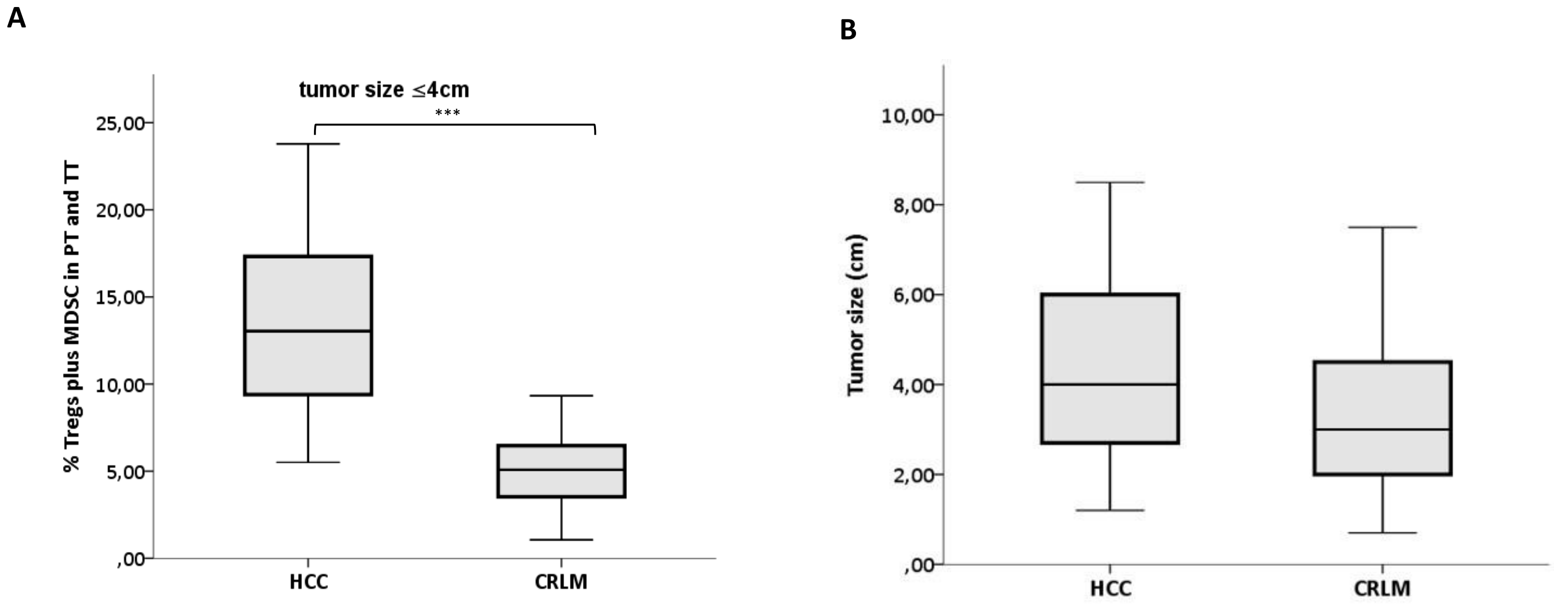

Supplementary Figure 10. Effect of CXCR4 inhibition in PB-HCC/CRLM derived Tregs

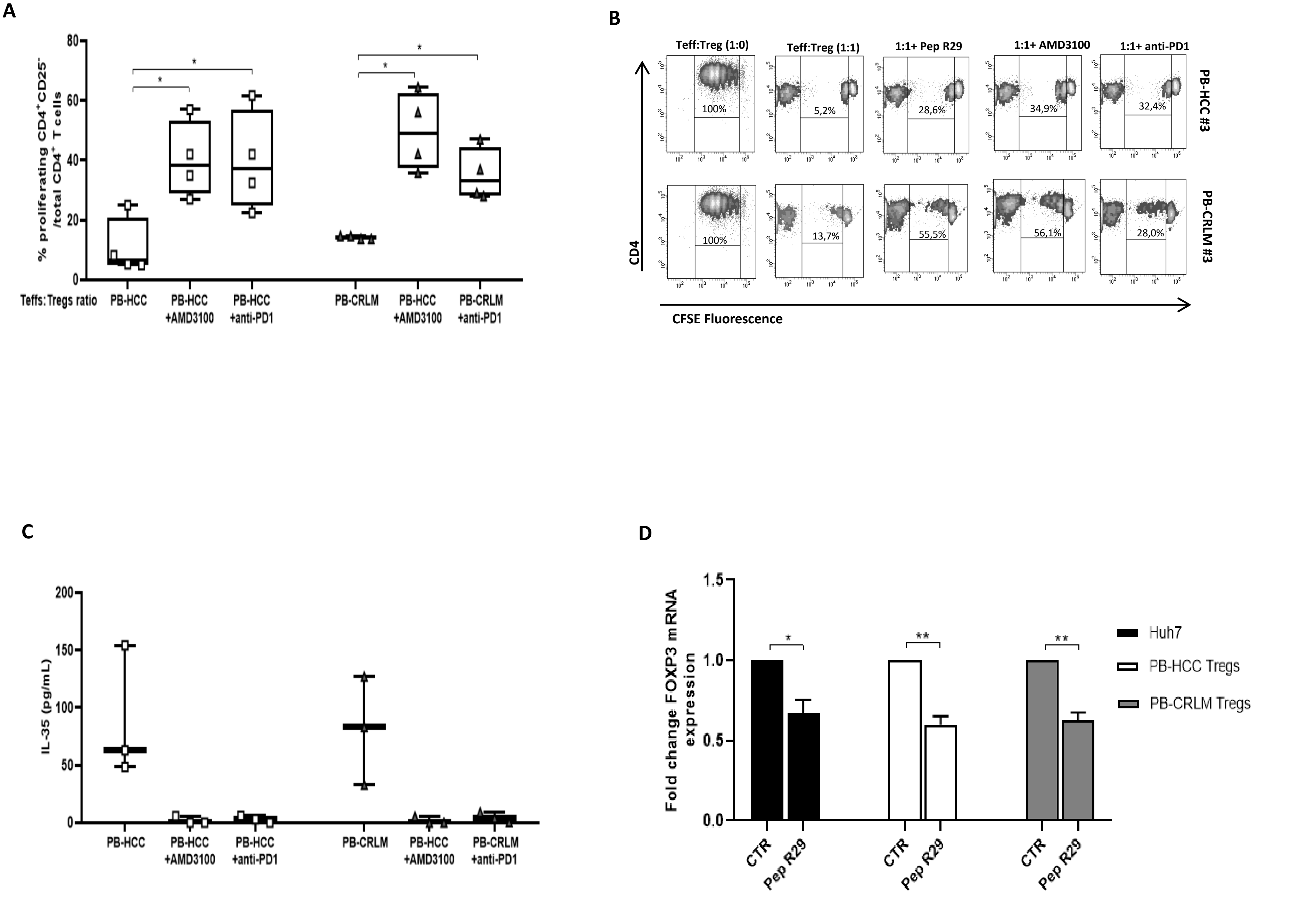

Supplement: Supplementary file 1 — Supplementary file1 (PDF 792 KB) [file 12072_2023_10537_MOESM1_ESM.pdf]
