# Supplementary material for: Hepatocellular carcinoma (HCC) tumor microenvironment is more suppressive than colorectal cancer liver metastasis (CRLM) tumor microenvironment
Source: Hepatol Int. 2023 May 4;18(2):568–81. doi: 10.1007/s12072-023-10537-6 (PMC11014815; doi:10.1007/s12072-023-10537-6)
Supplement: Supplementary file 2 — Supplementary file2 (DOCX 46 KB) [file 12072_2023_10537_MOESM2_ESM.docx]

|  | |  | **Gender** | **Age** | **Number of nodule** | **Size of nodule (cm)** | **Etiology** | | **Hepatic cirrhosis** | | **Grading** | | **Child-Pugh** | | **% CD4+CD25hiFOXP3+/total lymphocytes** | | | | | | | | |  |  |
| --- | --- | --- | --- | --- | --- | --- | --- | --- | --- | --- | --- | --- | --- | --- | --- | --- | --- | --- | --- | --- | --- | --- | --- | --- | --- |
|  |  | |  |  |  |  |  | |  | |  | |  | | **PB** | | | **PT** | | | | **TT** | | |  |
| **1.** | | ID 1 | M | 79 | 1 | 5.2 | HCV | | YES | | G2 | | A | | - | | | 0.57 | | | | 0.40 | | |  |
| **2.** | | ID 2 | M | 73 | 1 | 4.9 | HCV | | YES | | G3 | | B | | 1.39 | | | - | | | | - | | |  |
| **3.** | | ID 3 | M | 76 | 2 | 5.0 | HCV | | YES | | G2 | | A | | 0.97 | | | 1.27 | | | | 6.32 | | |  |
| **4.** | | ID 4 | M | 71 | 1 | 4.0 | HCV | | YES | | G2 | | - | | 1.30 | | | - | | | | - | | |  |
| **5.** | | ID 5 | F | 73 | 1 | 3.5 | HCV | | YES | | G2 | | A | | 1.21 | | | 0.70 | | | | - | | |  |
| **6.** | | ID 6 | F | 85 | 1 | 7.0 | HCV | | YES | | G2 | | A | | 0.77 | | | 6.20 | | | | 1.53 | | |  |
| **7.** | | ID 7 | M | 72 | 1 | 3.9 | HCV | | YES | | G2 | | A | | 1.23 | | | 2.03 | | | | 1.57 | | |  |
| **8.** | | ID 8 | M | 68 | 1 | 7.0 | HCV | | YES | | G2 | | A | | 1.67 | | | 0.37 | | | | 1.34 | | |  |
| **9.** | | ID 9 | M | 81 | 1 | 4.9 | HCV | | YES | | G3 | | A | | 1.42 | | | 1.02 | | | | 1.71 | | |  |
| **10.** | | ID 10 | F | 57 | 1 | 4.0 | HCV | | YES | | G2 | | A | | 0.78 | | | 0.49 | | | | 1.73 | | |  |
| **11.** | | ID 11 | M | 70 | 1 | 2.2 | HCV | | YES | | - | | A | | 2.64 | | | 0.20 | | | | 0.06 | | |  |
| **12.** | | ID 13 | M | 65 | 1 | 2.0 | HCV | | YES | | G2 | | B | | 0.40 | | | 0.44 | | | | 1.50 | | |  |
| **13.** | | ID 14 | M | 71 | 2 | 7.8 | HCV | | YES | | G2 | | B | | 0.38 | | | 0.78 | | | | 1.89 | | |  |
| **14.** | | ID 15 | F | 67 | 2 | 3.0 | HCV | | YES | | G2 | | A | | 2.46 | | | 2.18 | | | | 3.32 | | |  |
| **15.** | | ID 16 | M | 73 | 1 | 1.2 | HCV | | YES | | G2 | | B | | 2.77 | | | 2.98 | | | | 3.16 | | |  |
| **16.** | | ID 17 | M | 62 | 1 | 6.4 | HCV | | YES | | G2 | | A | | 1.33 | | | 6.07 | | | | 8.00 | | |  |
| **17.** | | ID 18 | M | 66 | 2 | 3.3 | HCV | | YES | | G2 | | A | | 0.20 | | | 0.70 | | | | 8.67 | | |  |
| **18.** | | ID 19 | F | 72 | 1 | 2.5 | HCV | | YES | | G2 | | A | | 0.48 | | | 0.46 | | | | 9.00 | | |  |
| **19.** | | ID 22 | F | 69 | 2 | 3.0 | HCV | | YES | | G2 | | A | | 0.85 | | | 0.60 | | | | 0.96 | | |  |
| **20.** | | ID 23 | M | 72 | 2 | 3.0 | HCV | | YES | | G2 | | A | | 0.82 | | | 2.60 | | | | - | | |  |
| **21.** | | ID 24 | M | 74 | 1 | 5.0 | HCV | | YES | | G2 | | A | | 0.68 | | | 0.23 | | | | 0.54 | | |  |
| **22.** | | ID 27 | M | 65 | 1 | 2.5 | HCV | | YES | | G2 | | A | | 0.15 | | | 0.29 | | | | 0.61 | | |  |
| **23.** | | ID 28 | F | 62 | 1 | 8.5 | HCV | | YES | | G3 | | - | | 0.11 | | | 0.10 | | | | 1.37 | | |  |
| **24.** | | ID 31 | M | 58 | 1 | 2.3 | HCV | | NO | | G3 | | - | | 1.41 | | | 0.27 | | | | 0.99 | | |  |
| **25.** | | ID 33 | F | 68 | 1 | 2.7 | HCV | | NO | | G2 | | - | | 0.90 | | | - | | | | - | | |  |
| **26.** | | ID 34 | M | 70 | 1 | 6.0 | HCV | | YES | | G2 | | B | | 2.48 | | | 0.59 | | | | 2.58 | | |  |
| **27.** | | ID 12 | M | 48 | 1 | 6.5 | HBV | | YES | | G2 | | A | | 0.39 | | | 0.72 | | | | 0.73 | | |  |
| **28.** | | ID 20 | M | 67 | 2 | 2.4 | HBV | | NO | | G2 | | A | | 0.88 | | | 0.94 | | | | 9.70 | | |  |
| **29.** | | ID 21 | M | 58 | 1 | 4.0 | NON-VIRAL | | YES | | G2 | | A | | 0.55 | | | 0.46 | | | | 2.63 | | |  |
| **30.** | | ID 26 | F | 53 | 1 | 8.0 | NON-VIRAL | | YES | | G2 | | A | | 1.21 | | | 0.65 | | | | 1.16 | | |  |
| **31.** | | ID 29 | M | 65 | 1 | 3.0 | NON-VIRAL | | NO | | G2 | | A | | 0.72 | | | - | | | | 2.13 | | |  |
| **32.** | | ID 30 | M | 78 | 1 | 8.0 | NON-VIRAL | | YES | | G2 | | - | | 0.40 | | | 0.40 | | | | 0.26 | | |  |
| **33.** | | ID 32 | F | 68 | 1 | 2.7 | NON-VIRAL | | NO | | G2 | | - | | 3.29 | | | - | | | | - | | |  |
| **34.** | | ID 25 | M | 57 | 1 | 4.0 | - | - | | G2 | | - | | 0.30 | | | - | | | 1.08 | | |  |  |  |
|  | |  |  |  |  |  |  | |  | |  | |  | | |  | | |  | |  | | | | |

*in gray 16 HCC patients with complete analysis (phenotype and T effector proliferation/ Treg dependent) in paired samples PB,

**Supplementary Table 1. Detailed characteristics of HCC patients**

**Supplementary Table 1. Detailed characteristics of HCC patients**

PT and TT.

**patients from ID 35 to ID 40 are not included in the detailed characteristics table as they were used for real-time analysis only.

|  |  |  |  | | | |  | | | |  | | | |  | | | |  | | | |  | | | |  | | |  | | | |  | | |  | |  |
| --- | --- | --- | --- | --- | --- | --- | --- | --- | --- | --- | --- | --- | --- | --- | --- | --- | --- | --- | --- | --- | --- | --- | --- | --- | --- | --- | --- | --- | --- | --- | --- | --- | --- | --- | --- | --- | --- | --- | --- |
|  |  |  | **Gender** | | **Age** | | | | **Number of nodule** | | | | **Size of nodule**  **(cm)** | | | | **CEA level (ng/mL)** | | | **Grading** | | | | | | **TNM** | | **% CD4+CD25hiFOXP3+/total lymphocytes** | | | | | | | | | | |  |
|  |  |  |  | | | |  | | | |  | | | |  | | | |  | | | |  | | | |  | | | **PB** | | **PT** | | | | | **TT** | | |
|  | **1.** | ID 8 | F | 81 | | | | 3 | | | | 3.0 | | | | - | | | | | G2 | | | T1N0M1 | | | | | 0.17 | | | | 3.32 | | | 1.41 | | | |
|  | **2.** | ID 14 | F | 83 | | | | 1 | | | | 9.0 | | | | 329.0 | | | | | G2 | | | T2N0M1 | | | | | 0.11 | | | | 2.05 | | | 3.08 | | | |
|  | **3.** | ID 15 | F | 47 | | | | 3 | | | | 4.6 | | | | 5.2 | | | | | G2 | | | T2N1M1 | | | | | 0.24 | | | | 0.50 | | | 1.01 | | | |
|  | **4.** | ID 5 | F | 52 | | | | >3 | | | | 4.0 | | | | 30.0 | | | | | G2 | | | T2N1M1 | | | | | 1.64 | | | | 1.24 | | | 2.20 | | | |
|  | **5.** | ID 31 | M | 61 | | | | 3 | | | | 3.0 | | | | 10.4 | | | | | G2 | | | T2N2M1 | | | | | 3.40 | | | | - | | | - | | | |
|  | **6.** | ID 13 | M | 56 | | | | 2 | | | | 10.0 | | | | 533.5 | | | | | G2 | | | T3N0M1 | | | | | 0.04 | | | | 0.94 | | | 5.30 | | | |
|  | **7.** | ID 23 | F | 52 | | | | 1 | | | | 1.0 | | | | 1.5 | | | | | G2 | | | T3N0M1 | | | | | 1.90 | | | | - | | | - | | | |
|  | **8.** | ID 27 | F | 67 | | | | 1 | | | | 3.5 | | | | 15.7 | | | | | G2 | | | T3N0M1 | | | | | 2.29 | | | | 2.53 | | | 2.58 | | | |
|  | **9.** | ID 1 | F | 61 | | | | 1 | | | | 3.0 | | | | 139.0 | | | | | G2 | | | T3N1M1 | | | | | 0.15 | | | | 0.50 | | | 1.03 | | | |
|  | **10.** | ID 3 | M | 63 | | | | 2 | | | | 7.5 | | | | 2.3 | | | | | G2 | | | T3N1M1 | | | | | 0.38 | | | | 0.46 | | | - | | | |
|  | **11.** | ID 4 | M | 65 | | | | 1 | | | | 10.0 | | | | 3.5 | | | | | G2 | | | T3N1M1 | | | | | 0.53 | | | | 0.20 | | | 2.81 | | | |
|  | **12.** | ID 6 | M | 66 | | | | 3 | | | | 2.5 | | | | 5.4 | | | | | G2 | | | T3N1M1 | | | | | 1.30 | | | | 1.32 | | | 1.76 | | | |
|  | **13.** | ID 9 | M | 47 | | | | 3 | | | | 2.0 | | | | 1.9 | | | | | G2 | | | T3N1M1 | | | | | 0.12 | | | | 0.11 | | | - | | | |
|  | **14.** | ID 10 | F | 63 | | | | >3 | | | | 2.6 | | | | 2.9 | | | | | G2 | | | T3N1M1 | | | | | 0.37 | | | | 0.11 | | | 5.45 | | | |
|  | **15.** | ID 11 | F | 59 | | | | 1 | | | | 3.3 | | | | 43.0 | | | | | G2 | | | T3N1M1 | | | | | 0.43 | | | | 0.57 | | | 2.28 | | | |
|  | **16.** | ID 12 | M | 68 | | | | >3 | | | | 0.8 | | | | 13.7 | | | | | G2 | | | T3N1M1 | | | | | 0.52 | | | | 0.61 | | | 1.89 | | | |
|  | **17.** | ID 18 | F | 69 | | | | 1 | | | | 2.8 | | | | 1.5 | | | | | G2 | | | T3N1M1 | | | | | 0.85 | | | | 0.47 | | | 1.04 | | | |
|  | **18.** | ID 24 | M | 48 | | | | 1 | | | | 3.0 | | | | 5.6 | | | | | G3 | | | T3N1M1 | | | | | 1.68 | | | | 0.59 | | | 0.47 | | | |
|  | **19.** | ID 22 | M | 46 | | | | 2 | | | | 2.8 | | | | 1.1 | | | | | G2 | | | T3NM1 | | | | | 3.17 | | | | 0.37 | | | 1.01 | | | |
|  | **20.** | ID 7 | M | 52 | | | | >3 | | | | 2.6 | | | | 6.8 | | | | | G2 | | | T4N1M1 | | | | | 1.06 | | | | 4.19 | | | 1.58 | | | |
|  | **21.** | ID 2 | M | 61 | | | | 1 | | | | 2.0 | | | | - | | | | | - | | | - | | | | | 0.20 | | | | 0.88 | | | - | | | |
|  | **22.** | ID 16 | F | 61 | | | | 3 | | | | 3.0 | | | | - | | | | | - | | | - | | | | | 1.23 | | | | 1.56 | | | 2.59 | | | |
|  | **23.** | ID 17 | F | 72 | | | | 2 | | | | 4.0 | | | | - | | | | | - | | | - | | | | | 2.07 | | | | 1.34 | | | 3.37 | | | |
|  | **24.** | ID 28 | M | 55 | | | | 2 | | | | 4.5 | | | | 4.0 | | | | | - | | | - | | | | | 0.96 | | | | - | | | - | | | |
| * in gray 12 CRLM patients with complete analysis (phenotype and T effector proliferation/ Treg dependent) in paired samples  PB, PT and TT.  **patient ID 19, 20, 21, 25, 26, 29, 30, 32, 33, 34 are not included in the detailed characteristics table as they were used for  real-time analysis only. |  |  |  | | |  | | | |  | | | |  | | | |  | | | |  | | |  | | | | | |  | | | |  | | |  |  |
|  |  |  |  | | | |  | | | |  | | | |  | | | |  | | | |  | | | |  | | |  | | | |  | | |  | |  |

**Supplemetary Table 2**. **Detailed characteristics of CRLM patients**

**Supplemetary Table 2. Detailed characteristics of 34 CRLM patients**
